# Organohalide-respiring *Desulfoluna* species isolated from marine environments

**DOI:** 10.1038/s41396-019-0573-y

**Published:** 2020-01-02

**Authors:** Peng Peng, Tobias Goris, Yue Lu, Bart Nijsse, Anna Burrichter, David Schleheck, Jasper J. Koehorst, Jie Liu, Detmer Sipkema, Jaap S. Sinninghe Damste, Alfons J. M. Stams, Max M. Häggblom, Hauke Smidt, Siavash Atashgahi

**Affiliations:** 1 Laboratory of Microbiology, Wageningen University & Research, Stippeneng 4, 6708 WE Wageningen, The Netherlands; 2 Department of Applied and Ecological Microbiology, Institute of Microbiology, Friedrich Schiller University, 07743 Jena, Germany; 3 College of Environmental Science and Engineering, Hunan University, 410082 Changsha, China; 4 Laboratory of Systems and Synthetic Biology, Wageningen University & Research, Stippeneng 4, 6708 WE Wageningen, The Netherlands; 5 Department of Biology, University of Konstanz, 78457 Konstanz, Germany; 6 The Konstanz Research School Chemical Biology, University of Konstanz, 78457 Konstanz, Germany; 7 Department of Biochemistry and Microbiology, Rutgers University, New Brunswick, NJ 08901 USA; 8 Department of Marine Microbiology and Biogeochemistry, NIOZ Royal Netherlands Institute for Sea Research, P.O. Box 59, 1790 AB Den Burg, The Netherlands; 9 Department of Earth Sciences, Faculty of Geosciences, Utrecht University, P.O. Box 80.121, 3508 TA Utrecht, The Netherlands; 10 Centre of Biological Engineering, University of Minho, Campus de Gualtar, 4710-057 Braga, Portugal; 11 Present Address: Department of Molecular Toxicology, Research Group Intestinal Microbiology, German Institute of Human Nutrition (DIfE),, Potsdam-Rehbrücke, Arthur-Scheunert-Allee 114-116, 14458 Nuthetal, Germany

**Keywords:** Microbiology, Environmental microbiology

## Abstract

The genus *Desulfoluna* comprises two anaerobic sulfate-reducing strains, *D. spongiiphila* AA1^T^ and *D. butyratoxydans* MSL71^T^, of which only the former was shown to perform organohalide respiration (OHR). Here we isolated a third strain, designated *D. spongiiphila* strain DBB, from marine intertidal sediment using 1,4-dibromobenzene and sulfate as the electron acceptors and lactate as the electron donor. Each strain harbors three reductive dehalogenase gene clusters (*rdhABC*) and corrinoid biosynthesis genes in their genomes, and dehalogenated brominated but not chlorinated organohalogens. The *Desulfoluna* strains maintained OHR in the presence of 20 mM sulfate or 20 mM sulfide, which often negatively affect other organohalide-respiring bacteria. Strain DBB sustained OHR with 2% oxygen in the gas phase, in line with its genetic potential for reactive oxygen species detoxification. Reverse transcription-quantitative PCR revealed differential induction of *rdhA* genes in strain DBB in response to 1,4-dibromobenzene or 2,6-dibromophenol. Proteomic analysis confirmed expression of *rdhA1* with 1,4-dibromobenzene, and revealed a partially shared electron transport chain from lactate to 1,4-dibromobenzene and sulfate, which may explain accelerated OHR during concurrent sulfate reduction. Versatility in using electron donors, *de novo* corrinoid biosynthesis, resistance to sulfate, sulfide and oxygen, and concurrent sulfate reduction and OHR may confer an advantage to marine *Desulfoluna* strains.

## Introduction

More than 5000 naturally produced organohalides have been identified, some of which have already been present in a variety of environments for millions of years [1]. In particular, marine environments are a rich source of chlorinated, brominated and iodinated organohalides produced by marine algae, seaweeds, sponges, and bacteria [2], Fenton-like [3] and photochemical reactions, as well as volcanic activities [4, 5]. Such a natural and ancient presence of organohalogens in marine environments may have primed development of various types of microbial dehalogenation [6]. Furthermore, marine environments and coastal regions in particular are also commonly reported to be contaminated with organohalogens from anthropogenic sources [7].

During organohalide respiration (OHR) organohalogens are used as terminal electron acceptors, and their reductive dehalogenation is coupled to energy conservation [8–10]. This process is mediated by reductive dehalogenases (RDases), which are membrane-associated, corrinoid-dependent, and oxygen-sensitive proteins [9–11]. The corresponding *rdh* gene clusters usually consist of *rdhA* encoding the catalytic subunit, *rdhB* encoding a putative membrane anchor protein [10], and a variable set of accessory genes encoding RdhC and other proteins likely involved in regulation, maturation and/or electron transport [12, 13]. The electron transport chain from electron donors to RDases has been classified into quinone-dependent (relying on menaquinones as electron shuttles between electron donors and RDases) and quinone-independent pathways [9, 10, 14]. Recent studies suggested that RdhC may serve as electron carrier during OHR in *Firmicutes* [15, 16].

OHR is mediated by organohalide-respiring bacteria (OHRB), which belong to a broad range of phylogenetically distinct bacterial genera. OHRB belonging to *Chloroflexi* or the genus *Dehalobacter* (*Firmicutes*, e.g., *Dehalobacter restrictus*) are specialists restricted to OHR, whereas proteobacterial OHRB and members of the genus *Desulfitobacterium* (*Firmicutes*, e.g., *Desulfitobacterium hafniense*) are generalists with a versatile metabolism [17, 18]. Numerous studies have reported OHR activity and occurrence of OHRB and *rdhA* genes in marine environments [6, 19–21]. Recent genomic [22–24] and single-cell genomic [25] analyses revealed widespread occurrence of *rdh* gene clusters in marine *Deltaproteobacteria*, indicting untapped potential for OHR. Accordingly, OHR metabolism was experimentally verified in three *Deltaproteobacteria* strains, not previously known as OHRB [23].

OHRB, and in particular members of the *Chloroflexi*, are fastidious microbes, and lack the ability to synthesize corrinoid co-factors *de novo* [9]. Moreover, many OHRB are susceptible to inhibition by oxygen [26], sulfate [27] or sulfide [28, 29]. For example, in the presence of both 3-chlorobenzoate and either sulfate, sulfite or thiosulfate, *Desulfomonile tiedjei* isolated from sewage sludge preferentially performed sulfur oxyanion reduction [30], and OHR inhibition was suggested to be caused by downregulation of *rdh* gene expression [30]. In contrast, concurrent sulfate reduction and OHR was observed in *Desulfoluna spongiiphila* AA1^T^ isolated from the marine sponge *Aplysina aerophoba* [20], and three newly characterized organohalide-respiring marine deltaproteobacterial strains [23]. Thus, sulfate- and sulfide-rich marine environments may have exerted a selective pressure resulting in development of sulfate- and sulfide-tolerant OHRB.

The genus *Desulfoluna* comprises two anaerobic sulfate-reducing strains, *D. spongiiphila* AA1^T^ isolated from the bromophenol-producing marine sponge *Aplysina aerophoba* [20, 31], and *D. butyratoxydans* MSL71^T^ isolated from estuarine sediments [32]. Strain AA1^T^ can reductively dehalogenate various bromophenols but not chlorophenols. The genome of strain AA1^T^ harbors three *rdhA* genes, one of which was shown to be induced by 2,6-dibromophenol (2,6-DBP) [21]. The OHR potential and the genome of strain MSL71^T^ have not been studied before. In this study, a third member of the genus *Desulfoluna*, designated *D. spongiiphila* strain DBB, was isolated from a marine intertidal sediment. The OHR metabolism of strains DBB and MSL71^T^ was verified in this study, providing further evidence for widespread OHR potential in marine Deltaproteobacteria [22–25]. Using in depth physiological, genomic and proteomic analyses, we aimed to unravel metabolic traits of these three strains, such as *de novo* corrinoid biosynthesis, resistance to sulfate, sulfide and oxygen, and versatility in using electron donors. Our results showed that resistance of *Desulfoluna* strains to sulfide was remarkable among the reported sulfate-reducing bacteria, and concurrent reduction of sulfate and organohalogens as terminal electron acceptors was unique among the currently known OHRB. Moreover, inability to dehalogenate organochlorines indicated niche specialization of the members of the genus *Desulfoluna* as chemoorganotrophic facultative OHRB in marine environments rich in sulfate and organobromines.

## Materials and methods

### Chemicals

Brominated, iodinated and chlorinated benzenes and phenols were purchased from Sigma-Aldrich. Other organic and inorganic chemicals used in this study were of analytical grade.

### Bacterial strains

*D. spongiiphila* AA1^T^ (DSM 17682^T^) and *D. butyratoxydans* MSL71^T^ (DSM 19427^T^) were obtained from the German Collection of Microorganisms and Cell Cultures (DSMZ, Braunschweig, Germany), and were cultivated as described previously [20, 32].

### Enrichment, isolation and cultivation of strain DBB

Surface sediment of an intertidal zone, predominantly composed of shore sediment, was collected at the shore in L’Escala, Spain (42°7'35.27"N, 3°8'6.99"E). Five grams of sediment was transferred into 120-ml bottles containing 50 ml of anoxic medium [33] with lactate and 1,4-dibromobenzene (1,4-DBB) as the electron donor and acceptor, respectively. Vitamin (without vitamin B_12_) and trace element solution was prepared as described previously [34]. The medium contained 10–30 g/L NaCl. Resazurin (0.005 g/L) and Na_2_S·9H_2_O (0.48 g/L) were added as redox indicator and reducing reagent, respectively. Sediment-free cultures were obtained by transferring the suspensions of the enrichment culture to fresh medium. A pure culture of a 1,4-DBB debrominating strain, designated as *D. spongiiphila* strain DBB, was obtained from a dilution series on solid medium with 0.8% low gelling agarose (congealing temperature 26–30 °C, Sigma-Aldrich, product number: A9414). A detailed description of enrichment, isolation and physiological characterization of strain DBB is provided in the Supplementary Information.

### DNA extraction and bacterial community analysis

DNA of the intertidal sediment (5 g) and the 1,4-DBB-respiring enrichment culture (10 ml) was extracted using the DNeasy PowerSoil Kit (MO-BIO, CA, USA). A 2-step PCR strategy was applied to generate barcoded amplicons from the V1—V2 region of bacterial 16S rRNA genes as described previously [35]. Primers for PCR amplification of the 16S rRNA genes are listed in Table S1. Sequence analysis was performed using NG-Tax [36]. Operational taxonomic units (OTUs) were assigned taxonomy using uclust [37] in an open reference approach against the SILVA 16S rRNA gene reference database (LTPs128_SSU) [38]. Finally, a biological observation matrix (biom) file was generated and sequence data were further analyzed using Quantitative Insights Into Microbial Ecology (QIIME) v1.2 [39].

### Genome sequencing and annotation

Genomic DNA of strains DBB and MSL71^T^ cells was extracted using the MasterPure™ Gram Positive DNA Purification Kit (Epicentre, WI, USA). The genomes were sequenced using the Illumina HiSeq2000 paired-end sequencing platform (GATC Biotech, Konstanz, Germany; now part of Eurofins Genomics Germany GmbH). The genome of strain DBB was further sequenced by PacBio sequencing (PacBio RS) to obtain longer read lengths. Optimal assembly kmer size for strain DBB was detected using kmergenie (v.1.7039) [40]. A *de novo* assembly with Illumina HiSeq2000 paired-reads was made with assembler Ray (v2.3.1) [40] using a kmer size of 81. A hybrid assembly for strain DBB with both the PacBio and the Illumina HiSeq reads was performed with SPAdes (v3.7.1, kmer size: 81) [41]. The two assemblies were merged using the tool QuickMerge (v1) [42]. Duplicated scaffolds were identified with BLASTN [43] and removed from the assembly. Assembly polishing was performed with Pilon (v1.21) [44] using the Illumina HiSeq reads. Optimal assembly kmer size for strain MSL71^T^ was also identified using kmergenie (v.1.7039), and a *de novo* assembly with Illumina HiSeq2000 paired-end reads was performed with SPAdes (v3.11.1) with a kmer-size setting of 79,101,117. FastQC and Trimmomatic (v0.36) [45] was used for read inspection and trimming using the trimmomatic parameters: TRAILING:20 LEADING:20 SLIDINGWINDOW:4:20 MINLEN:50. Trimmed reads were mapped with Bowtie2 v2.3.3.1 [46]. Samtools (v1.3.1) [47] was used for converting the bowtie output to a sorted and indexed bam file. The assembly was polished with Pilon (v1.21).

### Transcriptional analysis of the *rdhA* genes of *D. spongiiphila* DBB

Transcriptional analysis was performed using DBB cells grown with lactate (20 mM), sulfate (10 mM) and either 1,4-DBB (1 mM) or 2,6-DBP (0.2 mM). DBB cells grown with lactate and sulfate but without any organohalogens were used as control. Ten replicate microcosms were prepared for each experimental condition, and at each sampling time point, two microcosms were randomly selected and sacrificed for RNA isolation as described previously [48]. RNA was purified using RNeasy columns (Qiagen, Venlo, The Netherlands) followed by DNase I (Roche, Almere, The Netherlands) treatment. cDNA was synthesized from 200 ng total RNA using SuperScript™ III Reverse Transcriptase (Invitrogen, CA, USA) following manufacturer’s instructions. Primers for RT-qPCR assays were listed in Table S1. RT-qPCR assays were performed as outlined in Supplementary Information.

### Protein extraction and proteomic analysis

Triplicate 100 ml cultures of strain DBB grown with lactate (20 mM) and sulfate (10 mM) (LS condition) or with lactate (20 mM), sulfate (10 mM), and 1,4-DBB (100 µM) (LSD condition) were used for proteomic analysis. Cells were collected by centrifugation at 4500 × *g* for 20 min at 4 °C. The cells were then re-suspended in 1 ml 100 mM Tris-HCl buffer (pH 7.5) containing 10 µl protease inhibitor (Halt Protease Inhibitor Cocktail; Thermo Fisher Scientific, Rockford, USA). Cells were lysed by sonication using a Branson sonifier (Branson, CT, USA) equipped with a 3 mm tip by six pulses of 30 s with 30 s rest in between of each pulse. Cell debris was removed by centrifugation at 10,000 × *g* for 10 min at 4 °C. The protein concentration of the cell-free extracts (CFE) was determined using the Bradford assay [49]. The total-proteomics samples were purified by SDS-PAGE (see below) and the analyses were done as described by Burrichter et al. [50]. For total protein analysis, CFE corresponding to 200 µg of protein was mixed with SDS-PAGE loading dye (Roti-Load 1, Carl Roth, Karlsruhe, Germany) and loaded onto an SDS gel (4% acrylamide in the stacking and 12% in the resolving gel) until the proteins had just entered the resolving gel (without any separation); the Coomassie-stained total-protein bands were excised and then subjected to peptide fingerprinting-mass spectrometry (see below). For analysis of proteins associated to the membrane, the membrane fragments in the CFE were separated by ultracentrifugation at 104,000 × *g* for 35 min at 4 °C; the membrane pellet was solubilized in SDS-PAGE loading dye (Roti-Load 1, Carl Roth, Karlsruhe, Germany) and purified by SDS-PAGE as described above. The unresolved protein bands excised from SDS-PAGE gels were subjected to peptide fingerprinting-mass spectrometry with Dr. Andreas Marquardt at the Proteomics Centre of the University of Konstanz (https://www.biologie.uni-konstanz.de/proteomics-centre/) [50]. The samples were processed by in-gel reduction with dithiothreitol, alkylation with chloroacetamide and tryptic digest. Each sample was analyzed twice on a Orbitrap Fusion with EASY-nLC 1200 (Thermo Fisher Scientific) and tandem mass spectra were searched against an appropriate protein database (see below) of strain DBB using Mascot (Matrix Science, London, UK) and Proteome Discoverer V1.3 (Thermo Fisher Scientific) with “Trypsin” enzyme cleavage, static cysteine alkylation by chloroacetamide, and variable methionine oxidation [50]. The protein database was constructed from the annotated genome of strain DBB by in vitro translation of genes. Statistical analysis was performed using prostar proteomics [51]. Top three peptide area values were log2-transformed and normalized against all columns (column sums function from prostar proteomics). The values of proteins detected in at least two of the three replicates were differentially compared and tested for statistical significance. Missing values were imputed using the SLSA function of prostar, and hypothesis testing with a student’s *t* test was performed for LSD vs LS growth conditions. The *p* values were Benjamini–Hochberg corrected and proteins with *p* values below 0.05 and a log2 value of 1 or larger were considered statistically significantly up- or downregulated.

### Analytical methods

Halogenated benzenes and benzene were analyzed on a GC equipped with an Rxi-5Sil capillary column (Retek, PA, USA) and a flame ionization detector (GC-FID, Shimadzu 2010). Halogenated phenols and phenol were analyzed on a Thermo Scientific Accela HPLC System equipped with an Agilent Poroshell 120 EC-C18 column and a UV/Vis detector. Organic acids and sugars were analyzed using a ThermoFisher Scientific SpectraSYSTEM™ HPLC equipped with an Agilent Metacarb 67H column and RI/UV detectors. Sulfate, sulfite and thiosulfate were analyzed using a ThermoFisher Scientific Dionex™ ICS-2100 Ion Chromatography System equipped with a Dionex^TM^ Ionpac^TM^ AS17 IC column and a suppressed conductivity detector. Cell growth under sulfate-reducing conditions was determined by measuring OD_600_ using a WPA CO8000 cell density meter (Biochrom, Cambridge, UK). Cell growth of strain DBB during OHR and in absence of sulfate was determined by quantifying the 16S rRNA gene copy number using qPCR. Sulfide was measured by a photometric method using methylene blue as described previously [52].

### Strain and data availability

*D. spongiiphila* strain DBB was deposited at DSMZ under accession number DSM 104433. The 16S rRNA gene sequences of strain DBB were deposited in GenBank (accession numbers: MK881098—MK881099). The genome sequences of strains DBB and MSL71 were deposited in the European Bioinformatics Institute (accession number: GCA_902498735 (DBB), GCA_900699765 (MSL71^T^)). A list of proteins detected from strain DBB under LS and LSD growth conditions is available in Dataset S1.

## Results and discussion

### Enrichment of 1,4-DBB debrominating cultures and isolation of strain DBB

**Fig. 1:**
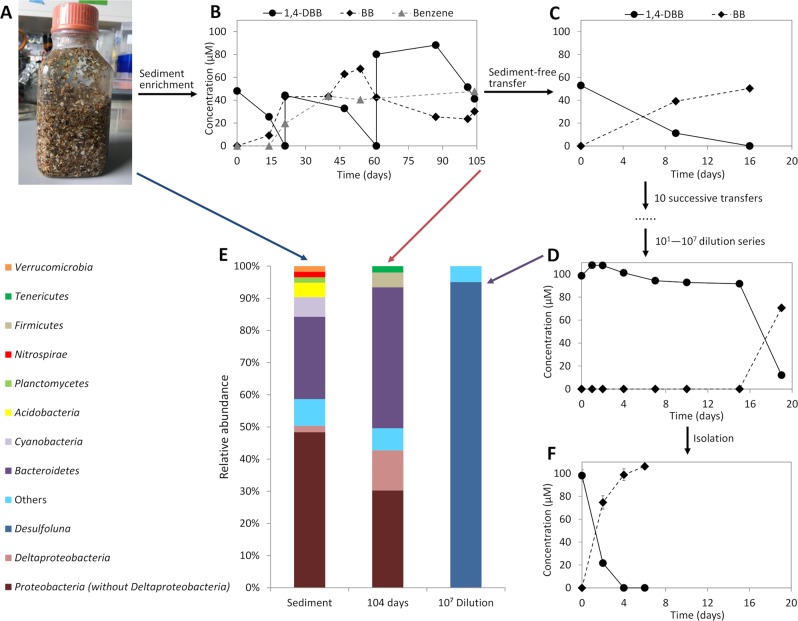
Enrichment and isolation of *D. spongiiphila* DBB. Intertidal sediment mainly composed of shore sediment used for isolation (**a**). Reductive debromination of 1,4-dibromobenzene (1,4-DBB) by: the original microcosms containing intertidal sediment (**b**), the sediment-free enrichment cultures (**c**), the most diluted culture (10^7^) in the dilution series (**d**). Phylogenetic analysis of bacterial communities in the microcosms from the shore sediment at time zero (left), the original 1,4-DBB debrominating enrichment culture after 104 days incubation (middle) and the 10^7^ dilution series culture (right) (**e**). Reductive debromination of 1,4-DBB to bromobenzene (BB) by the isolated pure culture (**f**). Sediment enrichment culture and sediment-free transfer cultures (**b**–**d**) were prepared in single bottles. Pure cultures (**f**) were prepared in duplicate bottles. Points and error bars represent the average and standard deviation of samples taken from the duplicate cultures. Phylogenetic data are shown at phylum level, except *Deltaproteobacteria* shown at class level and *Desulfoluna* at genus level. Taxa comprising less than 1% of the total bacterial community are categorized as ‘Others’.

Reductive debromination of 1,4-DBB to bromobenzene (BB) and benzene was observed in the original cultures containing intertidal sediment (Fig. 1a, b). Debromination of 1,4-DBB was maintained in the subsequent sediment-free transfer cultures (Fig. 1c). However, benzene was no longer detected and BB was the only debromination product, indicating loss of the BB-debrominating population. Up to date, the only known OHRB that can debrominate BB to benzene is *Dehalococcoides mccartyi* strain CBDB1 [53]. 1,4-DBB debromination to BB was stably maintained during subsequent transfers (data not shown) and after serial dilution (Fig. 1d). Bacterial community analysis showed an increase in the relative abundance of *Deltaproteobacteria* from ~2% in the intertidal sediment at time zero to ~13% after 104 days of enrichment (Fig. 1e). The genus *Desulfoluna* was highly enriched from below 0.1% relative abundance in the original sediment to more than 80% relative abundance in the most diluted culture (10^7^ dilution) (Fig. 1e).

Single colonies were observed in roll tubes with 0.8% low-melting agarose after 15 days of incubation. Among the six single colonies randomly selected and transferred to liquid media, one showed 1,4-DBB debromination (Fig. 1f) which was again subjected to the roll tube isolation procedure to ensure purity. The final isolated strain was designated strain DBB.

### Characterization of the *Desulfoluna* strains

Cells of strain DBB were slightly curved rods with a length of 1.5–3 µm and a diameter of 0.5 µm as revealed by SEM (Figs. S1A and S1B), which was similar to strain AA1^T^ (Fig. S1C) and MSL71^T^ (Fig. S1D). In contrast to strain AA1^T^ [20], but similar to strain MSL71^T^ [32], strain DBB was motile when observed by light microscopy, with evident flagella being observed by SEM (Fig. S1A, B).

The cellular fatty acid profiles of the three strains consisted mainly of even-numbered saturated and mono-unsaturated fatty acids (Table S2).

Strain DBB used lactate, pyruvate, formate, malate and butyrate as electron donors for sulfate reduction (Table 1). Lactate was degraded to acetate, which accumulated without further degradation, and sulfate was reduced to sulfide (Fig. S2A). In addition, sulfite and thiosulfate were utilized as electron acceptors with lactate as the electron donor (Table 1). Sulfate and 1,4-DBB could be concurrently utilized as electron acceptors by strain DBB (Fig. S2). Independent of the presence of sulfate in the medium, strain DBB stoichiometrically debrominated 1,4-DBB to bromobenzene (BB), and 2-bromophenol (2-BP), 4-bromophenol (4-BP), 2,4-bromophenol (2,4-DBP), 2,6-DBP, 2,4,6-tribromophenol (2,4,6-TBP), 2-iodophenol (2-IP) and 4-iodophenol (4-IP) to phenol (Table 1) using lactate as the electron donor. In the absence of sulfate, the growth yield of strain DBB was (8.6 ± 4.4) × 10^12^ 16S rRNA gene copies per mol bromide released from 1,4-DBB indicating energy conservation by reductive debromination. Hydrogen was not used as an electron donor for 1,4-DBB debromination or sulfate reduction (data not shown). Strain DBB was unable to dehalogenate the tested chlorinated aromatic compounds and several other bromobenzenes listed in Table 1. This is in accordance with the dehalogenating activity reported for strain AA1^T^ that was unable to use chlorinated aromatic compounds as electron acceptors [20]. The majority of the known organohalogens from marine environments are brominated [1] and hence marine OHRB may be less exposed to organochlorine compounds in their natural habitats. For instance, strain AA1^T^ was isolated from the marine sponge *Aplysina aerophoba* [20] in which organobromine metabolites can account for over 10% of the sponge dry weight [54].

**Table 1:** Physiological and genomic properties of *Desulfoluna* strains.

Strain	DBB	AA1^T a^	MSL71^T b^
Isolation source	Marine intertidal sediment	Marine sponge	Estuarine sediment
Cell morphology	Curved rods	Curved rods	Curved rods
Optimum NaCl concentration (%)	2.0	2.5	2.0
Temperature optimum/range (^o^C)	30/10–30	28/10–36	30/ND^c^
Utilization of electron donors			
Lactate	+	+	+
Butyrate	+	−	+
Formate	+	+	+
Acetate	−	−	−
Fumarate	−	−	−
Citrate	−	+	−
Glucose	−	+	−
Malate	+	+	+
Pyruvate	+	+	+
Hydrogen	−^d^	ND	+
Propionate	−	−	−
Succinate	−	−	−
Utilization of electron acceptors			
Sulfate	+	+	+
Sulfite	+	+	+
Thiosulfate	+	+	+
1,4-Dibromobenzene	+	+^e^	−^e^
1,2-Dibromobenzene	−	ND	ND
1,3-Dibromobenzene	−	ND	ND
1,2,4-Tribromobenzene	−	ND	ND
Bromobenzene	−	ND	ND
1,2-Dichlorobenzene	−	ND	ND
1,3-Dichlorobenzene	−	ND	ND
1,4-Dichlorobenzene	−	ND	ND
1,2,4-Trichlorobenzene	−	ND	ND
2-Bromophenol	+	+	+^e^
4-Bromophenol	+	+	−^e^
2,4-Dibromophenol	+	+	+^e, f^
2,6-Dibromophenol	+	+	+^e^
2,4,6-Tribromophenol	+	+	+^e, f^
2-Iodophenol	+	+^e^	−^e^
4-Iodophenol	+	+^e^	−^e^
2,4-Dichlorophenol	−	−	−^e^
2,6-Dichlorophenol	−	−	−^e^
2,4,6-Trichlorophenol	−	−	−^e^
Genomic information			
Genome size (Mb)	6.68	6.53^g^	6.05^h^
G+C content (%)	57.1	57.9^g^	57.2^h^
Total genes	5497	5356^g^	4894^h^
Total proteins	5301	5203^g^	4186^h^

^a^Data from Ahn et al. [20]

^b^Data from Suzuki et al. [32]

^c^ND not determined

^d^Tested with 1,4-dibromobenzene as the electron acceptor

^e^Data from this study

^f^4-Bromophenol rather than phenol was the debromination product

^g^Data from GenBank (accession number: NZ_FMUX01000001.1)

^h^Predicted based on draft genome

### Genomic and phylogenetic characterization of the *Desulfoluna* strains

The three *Desulfoluna* strains showed similar overall genome features (Table 1, Tables S3 and S4). The complete genome of strain DBB consists of a single chromosome with a size of 6.68 Mbp (Fig. S3). The genomes of strain AA1^T^ (GenBank accession number: NZ_FMUX01000001.1) and strain MSL71^T^ (sequenced in this study) are draft genomes with similar G + C content (Table 1). The average nucleotide identity (ANI) of the DBB genome to AA1^T^ and MSL71^T^ genomes was 98.5% and 85.9%, respectively. This indicates that DBB and AA1^T^ strains belong to the same species of *D. spongiiphila* [55]. 16S rRNA gene and protein domain-based phylogenetic analyses with other genera of the *Desulfobacteraceae* placed *Desulfoluna* strains in a separate branch of the corresponding phylogenetic trees (Fig. S4). Whole-genome alignment of strains DBB, AA1^T^ and MSL71^T^ revealed the presence of 11 locally colinear blocks (LCBs) with several small regions of inversion and rearrangement (Fig. S5). A site-specific recombinase gene (DBB_14420) was found in one of the LCBs. The same gene was also found in the corresponding inversed and rearranged LCBs in AA1^T^ (AA1_11599) and MSL71^T^ (MSL71_ 48620), suggesting a role of the encoded recombinase in genomic rearrangement in the *Desulfoluna* strains.

### Comparison of the *rdh* gene region of the *Desulfoluna* strains

**Fig. 2:**
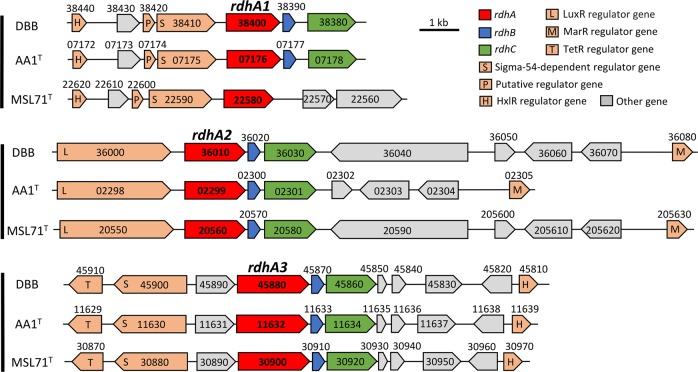
Comparison of the *rdh* gene clusters in *D. spongiiphila* DBB, *D. spongiiphila* AA1^T^ and *D. butyratoxydans* MSL71^T^. Numbers indicate the locus tags of the respective genes.

Similar to strain AA1^T^ [21], the genomes of strains DBB and MSL71^T^ also harbor three *rdhA* genes. The amino acid sequences of the RdhA homologs in DBB share >99% identity to the corresponding RdhAs in AA1^T^, and 80–97% identity with the corresponding RdhAs in MSL71^T^ (Fig. 2). However, the three distinct RdhA homologs in the *Desulfoluna* strains share low identity (20–30%) with each other, and they form three distant branches in the phylogenetic tree of RdhAs [18], and cannot be grouped with any of the currently known RdhA groups (Fig. S6). Therefore, we propose three new RdhA homolog groups, RdhA1 including DBB_38400, AA1_07176 and MSL71_22580; RdhA2 including DBB_36010, AA1_02299 and MSL71_20560, and RdhA3 including DBB_45880, AA1_11632 and MSL71_30900 (Fig. 2, Fig. S6).

The *rdh* gene clusters in DBB and MSL71^T^ show a similar gene order as the corresponding *rdh* gene clusters in AA1^T^ (Fig. 2), except that the *rdhA1* gene cluster of MSL71^T^ lacks *rdhB* and *rdhC*. Genes encoding sigma-54-dependent transcriptional regulators in the *rdhA1* and *rdhA3* gene clusters of AA1^T^ [21] are also present in the corresponding gene clusters of DBB and MSL71^T^ (Fig. 2). Likewise, genes encoding the LuxR and MarR-type regulators are present up- and downstream of the *rdhA2* gene clusters of DBB and MSL71^T^, in line with the organization of the *rdhA2* gene cluster of AA1^T^ (Fig. 2). This may indicate similar regulation systems of the *rdh* genes in the *Desulfoluna* strains studied here. The conserved motifs from known RDases (RR, C1−C5, FeS1, and FeS2) [56, 57] are also conserved among all the RdhAs of the *Desulfoluna* strains, except for RdhA1 of MSL71^T^, which lacks the RR motif (Fig. S7). This may indicate a cytoplasmic localization and a non-respiratory role of RdhA1 in strain MSL71^T^ [6].

### OHR metabolism of *D. butyratoxydans* MSL71^T^

Guided by the genomic potential of strain MSL71^T^ for OHR, physiological experiments in this study confirmed that strain MSL71^T^ is indeed capable of using 2-BP, 2,4-DBP, 2,6-DBP and 2,4,6-TBP as electron acceptors with lactate as the electron donor. Similar to DBB and AA1^T^, chlorophenols such as 2,4-DCP, 2,6-DCP and 2,4,6-TCP were not dehalogenated by strain MSL71^T^ (Table 1). In contrast to strains DBB and AA1^T^, strain MSL71^T^ was unable to debrominate 1,4-DBB and 4-BP. Hence, debromination of 2,4-DBP and 2,4,6-TBP was incomplete with 4-BP as the final product rather than phenol (Table 1). Moreover, strain MSL71^T^ was unable to deiodinate 2-IP and 4-IP, again in contrast to strains DBB and AA1^T^ (Fig. S8, Table 1).

### Induction of *rdhA* genes during OHR by strain DBB

**Fig. 3:**
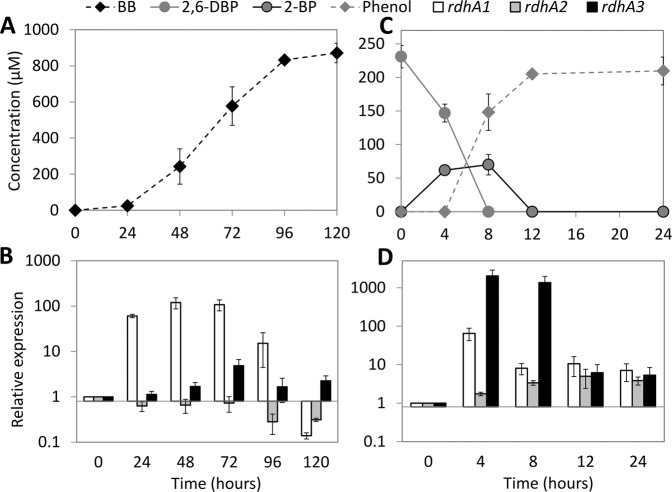
Differential induction of *rdhA* genes during 1,4-DBB and 2,6-DBP debromination by *D. spongiiphila* DBB. Debromination of 1,4-DBB (**a**) and 2,6-DBP (**c**) by strain DBB and RT-qPCR analysis of relative induction of its three *rdhA* genes during debromination of 1,4-DBB (**b**) and 2,6-DBP (**d**). Error bars in panels **a** and **c** indicate the standard deviation of two random cultures analyzed out of 10 replicates. The concentration of 1,4-DBB (>0.1 mM) could not be accurately measured due to large amount of undissolved compound and hence was not plotted. Error bars in panels **b** and **d** indicate standard deviation of triplicate RT-qPCRs performed on samples withdrawn from duplicate cultures at each time point (*n* = 2 × 3).

When strain DBB was grown with sulfate and 1,4-DBB with concomitant production of BB (Fig. 3a), its *rdhA1* gene showed significant up-regulation (60-fold) at 24 h, reached its highest level (120-fold) at 48–72 h, and then decreased (Fig. 3b). In contrast, no significant up-regulation of *rdhA2* or *rdhA3* was noted, suggesting that RdhA1 mediates 1,4-DBB debromination. Accordingly, RdhA1 was found in the proteome of the LSD growth condition but not in that of the LS condition (Table S5, Datasets S2, S3). When strain DBB was grown with sulfate and 2,6-DBP, both *rdhA1* and *rdhA3* were significantly up-regulated and reached their highest level at 4 h (65- and 2000-fold, respectively, Fig. 3d). However, *rdhA3* was the dominant gene at 8 h (Fig. 3d), after which 2-BP was debrominated to phenol (Fig. 3c), indicating a role of RdhA3 in 2,6-DBP and 2-BP debromination by strain DBB. A previous transcriptional study of the *rdhA* genes in strain AA1^T^ during 2,6-DBP debromination also showed a similar induction of its *rdhA3* [21].

### Corrinoid biosynthesis in *Desulfoluna* strains

**Fig. 4:**
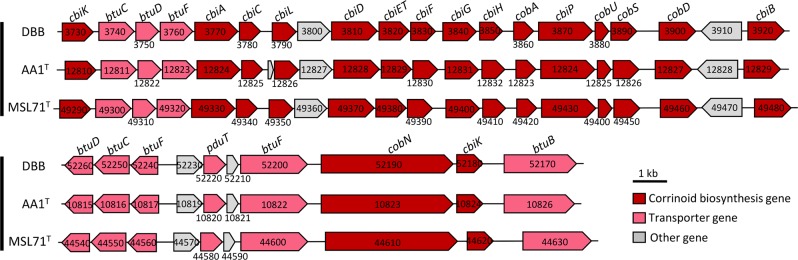
Corrinoid biosynthesis and transporter gene clusters of *Desulfoluna* strains. Numbers indicate the locus tags of the respective genes. The corresponding enzymes encoded by the genes and their functions in corrinoid biosynthesis are indicated in Table S4.

Most known RDases depend on corrinoid cofactors such as cyanocobalamin for dehalogenation activity [10]. Both strains DBB (this study) and AA1^T^ [21] were capable of OHR in the absence of externally added cobalamin. With one exception (*cbiJ*), the genomes of the *Desulfoluna* strains studied here harbor all genes necessary for *de novo* anaerobic corrinoid biosynthesis starting from glutamate (Table S6). The genes for cobalamin biosynthesis from precorrin-2 are arranged in one cluster (DBB_3730–3920, AA1_12810–12829, MSL71_49290–49480) including an ABC transporter (*btuCDF*) for cobalamin import (Fig. 4). Three of the proteins encoded by DBB_3730–3920 (Cbik: 3730, CbiL: 3790, CbiH: 3850) could be quantified in the proteome of cells grown under both the LS and LSD conditions, whereas CobH/CbiC (3780) and CobU (3880) could be quantified for LSD and LS conditions, respectively (Table S5, Datasets S2, S3). The abundance of the cobalamin biosynthesis proteins was not significantly different between LS and LSD conditions (Table S5, Datasets S2, S3), except for the tetrapyrrole methylase CbiH encoded by DBB_3850 that was significantly more abundant in LSD cells (Table S5, Dataset S3). The detection of cobalamin biosynthesis proteins in the absence of 1,4-DBB in LS condition could be due to the synthesis of corrinoid-dependent enzymes in the absence of an organohalogen. Accordingly, three corrinoid-dependent methyltransferase genes (encoded by DBB_7090, 43520, 16050) were detected in the proteomes, which might be involved in methionine, methylamine or *O*-demethylation metabolism. This might also indicate a constitutive expression of the corresponding genes, in contrast to the organohalide-induced cobalamin biosynthesis in *Sulfurospirillum multivorans* [58].

### Sulfur metabolism and impact of sulfate and sulfide on debromination by *Desulfoluna* strains

**Fig. 5:**
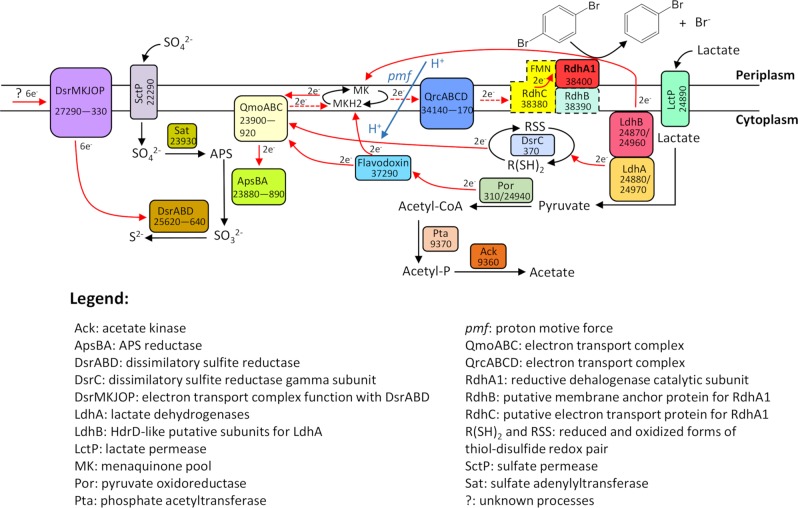
Preliminary electron transport pathway scheme based on the genomic and proteomic analysis of *D. spongiiphila* DBB grown on lactate, sulfate and 1,4-DBB (LSD condition). Corresponding gene locus tags are given for each protein. Proteins shown in dashed line square were not detected under the tested conditions. Probable electron flow path is shown in red arrows, and the dashed red arrows indicate reverse electron transport. The *pmf* is built up by ATPase using ATP generated by substrate-level phosphorylation via Por, Pta and Ack. Note that the distribution of electrons to the electron transport chains is not equal between sulfate respiration and OHR, but shifted heavily toward sulfate respiration due to excess sulfate (20 mM vs. 100 µM 1,4-DBB).

All three strains were capable of using sulfate, sulfite, and thiosulfate as terminal electron acceptors (Table 1). Among the four sulfate permeases encoded in the genomes of the *Desulfoluna* strains (Table S7), one (DBB_22290) was detected in DBB cells grown under LS and LSD conditions (Table S5, Dataset, S3). The genes involved in sulfate reduction, including those encoding sulfate adenylyltransferase (Sat), APS reductase (AprBA) and dissimilatory sulfite reductase (DsrAB), were identified in the genomes of all three strains (Table S7). The corresponding proteins were detected in DBB cells grown under both LS and LSD conditions (Fig. 5, Table S5) with AprBA, disulfite reductase (DsrMKJOP) and Sat among the most abundant proteins in both, soluble and membrane fractions (Datasets S2, S3). Interestingly, thiosulfate reductase genes were not found in any of the three genomes, whereas all strains can use thiosulfate as the electron acceptor (Table 1). *Desulfitobacterium metallireducens* was also reported to reduce thiosulfate despite lacking a known thiosulfate reductase gene [59, 60], suggesting the existence of a not-yet-identified gene encoding a thiosulfate reductase [60].

Sulfate and sulfide are known inhibitors for many OHRB [30, 61, 62]. However, debromination of 2,6-DBP was not affected in *Desulfoluna* strains in the presence of up to 20 mM sulfate (Fig. S9B, D, F), and sulfate and 2,6-DBP were reduced concurrently (Fig. S9). This is similar to some other *Deltaproteobacteria* [23], but in contrast to *D. tiedjei* which preferentially performs sulfate reduction over OHR with concomitant downregulation of *rdh* gene expression [30]. Moreover, sulfide, an RDase inhibitor in *D. tiedjei* [63] and *Dehalococcoides mccartyi* strains [28, 29], did not impact 2,6-DBP debromination by *Desulfoluna* strains even at a concentration of 10 mM (Fig. S10A–F). However, debromination was delayed in the presence of 20 mM sulfide, and no debromination was noted in the presence of 30 mM sulfide (Fig. S10G–L). This high resistance to sulfide was not reported before for the known OHRB, and is also rare among sulfate-reducing bacteria [64], and may confer an ecological advantage to these sulfate-reducing OHRB.

### Electron transport chains of strain DBB

Based on previous studies with *Desulfovibrio vulgaris* Hildenborough and *Desulfovibrio alaskensis* G20 that are phylogenetically related to *Desulfoluna* (Fig. S4), the following electron transport pathway in strain DBB with lactate and sulfate can be proposed (Fig. 5): the two Ldhs either reduce menaquinone directly, or transfer electrons via their HdrD-like subunit LdhB and DsrC (a high redox potential electron carrier with disulfide/dithiol (RSS/R(SH)_2_)) to QmoA [65, 66]. The pyruvate produced by lactate oxidation is further oxidized by Por, and the released electrons are carried/transferred by a flavodoxin, which is a likely candidate for a catabolic electron carrier as suggested by its high abundance in our proteome analysis (Table S5). The electrons from the low-potential flavodoxin could either be transferred to menaquinone, or confurcated to QmoABC together with the electrons from the high-potential (disulfide bond) DsrC. QmoABC then reduces menaquinone (Fig. 5), and the electrons are subsequently transferred from QmoABC to the APS reductase (ApsBA) which is, together with three other enzyme complexes (Sat, DsrABD, and DsrMKJOP), responsible for the sulfate reduction cascade [67].

Electron transport from QmoABC to RdhA via menaquinol needs to overcome an energy barrier because electron transport from menaquinol (*E*^0^’ = −75 mV) to the RDase (*E*^0^’ (CoII/CoI) ≈ −360 mV) is thermodynamically unfavorable [10]. However, the protein(s) and process(es) involved to overcome this energy barrier is not clear. One possibility is reverse electron transport as shown for *D. vulgaris* Hildenborough and *D. alaskensis* G20 that transfer electrons derived from lactate oxidation through menaquinol to a periplasmic type I cytochrome *c*3 (TpIc3, *E*^0^’ = −325 to −170 mV) during syntrophic growth [65]. The energy required for this reverse electron transport is generated by the proton motive force (*pmf*) mediated by the Qrc complex [68]. Strain DBB might use a similar strategy to overcome the energy barrier to transfer electrons from menaquinol to the periplasmic RdhA1 (Fig. 5). Qrc was detected in the proteome of DBB cells grown under both LS and LSD conditions (Table S5), whereas the TpIc3 was not identified in the *Desulfoluna* genomes. Instead of TpIc3, strain DBB could use RdhC1, a homolog to PceC of *Dehalobacter restrictus* that was proposed to mediate electron transfer from menaquinol to PceA via its exocytoplasmic-facing flavin mononucleotide (FMN) co-factor [16]. Similar to *D. restrictus*, the RdhC1 of strain DBB contains a conserved FMN binding motif (in particular the fully conserved threonine residue) and two CX_3_CP motifs predicted to have a role in electron transfer [16] (Fig. S11). In addition, five transmembrane helices of RdhC1 in strain DBB are also conserved (Fig. S12), indicating a similar function of RdhC1 in electron transfer from menaquinones to RdhA1 via FMN co-factor (Fig. 5). However, since RdhC was not detected in our proteome analysis likely due to tight interactions with the membrane with its five transmembrane helixes, further biochemical studies are necessary to verify the proposed role of RdhC1 in *Desulfoluna* OHR.

The *pmf* derived from sulfate reduction might be used for reverse electron transport during OHR, which may explain accelerated OHR with concurrent sulfate reduction (Fig. S2). Further studies such as construction of *Desulfoluna* mutant strains lacking *qrc* genes are necessary to verify the function of Qrc in energy metabolism of *Desulfoluna*.

### Potential oxygen defense in *Desulfoluna* strains

Sulfate reducers, which have been assumed to be strictly anaerobic bacteria, not only survive oxygen exposure but can also utilize it as an electron acceptor [69, 70]. However, the response of organohalide-respiring sulfate reducers to oxygen exposure is not known. Most of the described OHRB are strict anaerobes isolated from anoxic and usually organic matter-rich subsurface environments [17]. In contrast, strain DBB was isolated from marine intertidal sediment mainly composed of shore sand (Fig. 1a), where regular exposure to oxic seawater or air can be envisaged. The genomes of the *Desulfoluna* strains studied here harbor genes encoding enzymes for oxygen reduction and reactive oxygen species (ROS) detoxification (Table S8). Particularly, the presence of a cytochrome *c* oxidase encoding gene is intriguing and may indicate the potential for oxygen respiration. Accordingly, in the presence of 2% oxygen in the headspace of DBB cultures, the redox indicator resazurin in the medium turned from pink to colorless within two hours, indicating consumption/reduction of oxygen by strain DBB. Growth of strain DBB on lactate and sulfate was retarded in the presence of 2% oxygen (Fig. S13C). However, in both the presence (Fig. S13C) and absence of sulfate (Fig. S13D), slower but complete debromination of 2,6-DBP to phenol was achieved with 2% oxygen in the headspace. Neither growth nor 2,6-DBP debromination was observed with an initial oxygen concentration of 5% in the headspace (Fig. S13E, F). Such resistance of marine OHRB to oxygen may enable them to occupy niches close to halogenating organisms/enzymes that nearly all use oxygen or peroxides as reactants [71]. For instance, the marine sponge *A. aerophoba* from which *D. spongiiphila* AA1^T^ was isolated [20] harbors bacteria with a variety of FADH_2_-dependent halogenases [72], and produces a variety of brominated secondary metabolites [54].

## Conclusions

Widespread environmental contamination with organohalogen compounds and their harmful impacts to human and environmental health has been the driver of chasing OHRB since the 1970s. In addition, the natural environment is an ample and ancient source of organohalogens, and accumulating evidence shows widespread occurrence of putative *rdhA* in marine environments [6, 24, 73–75]. The previous isolation and description of strain AA1^T^ from a marine sponge, the isolation of strain DBB from intertidal sediment samples, and verification of the OHR potential of strain MSL71^T^ in this study indicate niche specialization of the members of the genus *Desulfoluna* as chemoorganotrophic facultative OHRB in marine environments. As such, *de novo* corrinoid biosynthesis, resistance to sulfate, sulfide and oxygen, versatility in using electron donors, respiration of brominated but not chlorinated aromatic compounds, and the capacity for concurrent sulfate and organohalogen respiration confer an advantage to *Desulfoluna* strains in marine environments rich in sulfate and organobromines.

## Supplementary information

Supplementary Information

Dataset 1

Dataset 2

Dataset 3
