# Supplementary material for: Organohalide-respiring Desulfoluna species isolated from marine environments
Source: ISME J. 2020 Jan 2;14(3):815–27. doi: 10.1038/s41396-019-0573-y (PMC7031245; doi:10.1038/s41396-019-0573-y)
Supplement: Supplementary file 1 — Supplementary Information [file 41396_2019_573_MOESM1_ESM.docx]

**Supplementary Information**

**Title: Organohalide-respiring *Desulfoluna* species isolated from marine environments**

**Running title: Organohalide-respiring *Desulfoluna* species**

Peng Peng,^a^ Tobias Goris,^b#^ Yue Lu,^c^ Bart Nijsse,^d^ Anna Burrichter,^i,j^ David Schleheck,^i,j^ Jasper J. Koehorst,^d^ Jie Liu,^h^ Detmer Sipkema,^a^ Jaap S. Sinninghe Damste,^e,f^ Alfons J. M. Stams,^a,g^ Max M. Häggblom,^h^ Hauke Smidt,^a^ Siavash Atashgahi^a*^

^a^ Laboratory of Microbiology, Wageningen University & Research, Stippeneng 4, 6708 WE Wageningen, The Netherlands

^b^ Department of Applied and Ecological Microbiology, Institute of Microbiology, Friedrich Schiller University, 07743 Jena, Germany

^c^ College of Environmental Science and Engineering, Hunan University, 410082 Changsha, China

^d^ Laboratory of Systems and Synthetic Biology, Wageningen University & Research, Stippeneng 4, 6708 WE Wageningen, The Netherlands

^e^ Department of Marine Microbiology and Biogeochemistry, NIOZ Royal Netherlands Institute for Sea Research, P.O. Box 59, 1790 AB Den Burg, The Netherlands

^f^ Department of Earth Sciences, Faculty of Geosciences, Utrecht University, P.O. Box 80.121, 3508 TA Utrecht, The Netherlands

^g^ Centre of Biological Engineering, University of Minho, Campus de Gualtar, 4710-057 Braga, Portugal

^h^ Department of Biochemistry and Microbiology, Rutgers University, New Brunswick, NJ 08901, USA

^i^ Department of Biology, University of Konstanz, 78457 Konstanz, Germany

^j^ The Konstanz Research School Chemical Biology, University of Konstanz, 78457 Konstanz, Germany

^*^Address correspondence to Siavash Atashgahi: [siavash.atashgahi@wur.nl](mailto:siavash.atashgahi@wur.nl)

^#^Present address: Department of Molecular Toxicology, Research Group Intestinal Microbiology, German Institute of Human Nutrition (DIfE), Potsdam-Rehbrücke, Arthur-Scheunert-Allee 114-116, 14458 Nuthetal

**This file includes:**

Supplementary Materials and Methods

Supplementary Figures S1 to S13

Supplementary Tables S1 to S8

References

**Supplementary Materials and Methods**

**Enrichment, isolation and cultivation of strain DBB**

The sediment sampling bottles were filled with seawater to leave no headspace. For preparation of microcosms, sediment (5 g) was transferred into 120 ml bottles containing 50 ml of anoxic medium ([1](#_ENREF_1)) and N_2_/CO_2_ (80 : 20%, 140 kPa) as the headspace. Vitamins and trace elements were added as described previously ([2](#_ENREF_2)) except that cyanocobalamin was omitted. Lactate (5 mM) and 1,4-dibromobenzene (1,4-DBB, 50 μM) were used as the electron donor and acceptor, respectively. 1,4-DBB was added from a 10 mM stock solution dissolved in acetone. The bottles were sealed with viton stoppers and aluminium crimp caps and incubated statically in the dark at 25°C. When 1,4-DBB debromination was stable in the transfer cultures (two days for debromination of 50 μM 1,4-DBB, after debromination of three spikes of 1,4-DBB), sediment-free cultures were obtained by transferring the suspensions of the enrichment culture (10% v/v) to fresh medium using the same growth condition as described above. After ten successive transfers of the sediment-free cultures, a dilution series from 10^1^ to 10^7^-fold was performed. The most diluted culture showing 1,4-DBB debromination (10^7^-fold) was then serially diluted from 10^1^- to 10^3^-fold in 25 ml roll tubes containing 10 ml medium and 0.8% low-melting point agarose (Sigma-Aldrich) and incubated in the dark at 25°C. Individual colonies were randomly picked and transferred into liquid medium to check for 1,4-DBB debromination. A culture showing debromination activity was re-isolated in roll tubes as described above to ensure the purity.

The optimum NaCl concentration for growth of strain DBB was determined in the range from 10 to 30 g/L. Using the optimal NaCl concentration (20 g/L), the following halogenated aromatic compounds were tested as electron acceptors for strain DBB with lactate (5 mM) as the electron donor and carbon source: 1,2-dibromobenzene (1,2-DBB), 1,3-dibromobenzene (1,3-DBB), 1,2,4-tribromobenzene (1,2,4-TBB), 2-bromophenol (2-BP), 4-bromophenol (4-BP), 2,4-dibromophenol (2,4-DBP), 2,6-dibromophenol (2,6-DBP), 2,4,6-tribromophenol (2,4,6-TBP), 2-iodophenol (2-IP), 4-iodophenol (4-IP), 1,2-dichlorobenzene (1,2-DCB), 1,3-dichlorobenzene (1,3-DCB), 1,4-dichlorobenzene (1,4-DCB), 1,2,4-trichlorobenzene (1,2,4-TCB), 2,4-dichlorophenol (2,4-DCP), 2,6-dichlorophenol (2,6-DCP) and 2,4,6-trichlorophenol (2,4,6-TCP). Brominated and chlorinated benzenes, 2,4,6-TBP and 2,4,6-TCP were added from 10 mM stock solutions dissolved in acetone to nominal concentrations of 100 μM in the medium. The remaining di- and mono-brominated phenols were added from 10 mM stock solutions in 0.1 N NaOH to nominal concentrations of 50—100 μM. Sulfate, sulfite and thiosulfate (5 mM) were tested as electron acceptors with 10 mM lactate as the electron donor. To test the utilization of electron donors, acetate, propionate, fumarate, malate, butyrate, lactate, pyruvate, succinate, glucose and citrate were added separately at 10 mM to the medium containing 10 mM sulfate. Utilization of formate (5 mM) as the electron donor for debromination of 1,4-DBB (100 µM) was tested in presence of acetate (5 mM) as the carbon source. Utilization of hydrogen (20 mM) as the electron donor for sulfate (5 mM) reduction and/or debromination of 1,4-DBB (100 µM) was tested in presence of acetate (5 mM) as the carbon source. To study the effect of sulfate and sulfide on debromination, sulfate (10—20 mM) or sulfide (1—30 mM) together with lactate (20—40 mM) were added to the medium containing 100 μM of 1,4-DBB or 2,6-DBP. To test the impact of oxygen on debromination, strain DBB was grown in medium without Na_2_S as the reducing agent, in presence or absence of sulfate (10 mM). The medium contained 20 mM lactate, 100 µM 2,6-DBP and 0%, 2% or 5% oxygen in the headspace.

**Cell morphology and cellular fatty acids analyses**

Cell morphology and motility were observed using a LEICA DM 2000 Microscope and a JEOL-6480LV Scanning Electron Microscope (SEM). Actively growing cells were directly observed under the 100x magnification objective of the LEICA DM 2000 Microscope. Sample fixation and dehydration for SEM were performed as described previously ([3](#_ENREF_3)). The cellular fatty acid composition was analysed from 500 ml cultures of AA1^T^, DBB and MSL71^T^, which were grown with 20 mM lactate and 10 mM sulfate. Fatty acids in the cell were analysed by acid hydrolysis of total cell material following a method previously described ([4](#_ENREF_4)). Briefly, the cultures were harvested at the early stationary growth phase by centrifugation at 4,700 × *g* for 15 min at 4°C. Cellular fatty acids were analysed by acid hydrolysis of total cell material following a method previously described ([4](#_ENREF_4)). The fatty acids were identified by analysis with gas chromatography-mass spectrometry before and after derivatisation of double bonds with dimethyl disulphide to enable localization of the double bond position ([4](#_ENREF_4)).

**Phylogenetic analysis**

A total of 21 sulfate-reducing Deltaproteobacterial genomes were selected for phylogenetic analysis of the *Desulfoluna* strains (Table S4). Phylogenetic analysis was conducted based on 16S rRNA gene sequences and protein domain as described previously ([5](#_ENREF_5), [6](#_ENREF_6)).

**RT-qPCR assays**

Primers for amplification of the three *rdhA* genes in strain DBB were designed using the NCBI online primer design tool (<http://www.ncbi.nlm.nih.gov/tools/primer-blast/>) (Table S1). In order to prepare standards for the qPCR assays, the *rdhA* genes were PCR amplified using the following program: 95°C for 5 min, followed by 30 cycles of 95°C for 30 s, 55°C for 30 s and 72°C for 30 s, followed by a final extension at 72°C for 10 min. The *rdhA* genes were then cloned into pGEM^®^-T Easy Vector (Promega, WI, USA) and introduced into *E. coli* JM109 competent cells (Promega, WI, USA). Plasmid purification and preparation of the dilution series of the RT-qPCR standards (from 10^1^ to 10^8^ copies/µl) were done as described earlier ([7](#_ENREF_7)). RT-qPCRs were performed using the iQ SYBR Green supermix (Bio-Rad, CA, USA). The RT-qPCR program was: 95°C for 10 min, followed by 40 cycles of 95°C for 15 s, 60°C for 30 s and 72°C for 30 s. Melting curves were measured from 65°C to 95°C with increments of 0.5°C and 10 s at each step. Transcription of the *rdhA* genes was determined using cDNA as the template. The transcript levels were calculated by relative quantification using the 2^-ΔΔCq^ method with the 16S rRNA gene as the reference gene ([8](#_ENREF_8), [9](#_ENREF_9)). Gene expression data was normalized to values observed at the 0 h time point, at which 1,4-DBB or 2,6-DBP were initially amended ([8](#_ENREF_8)). A relative expression difference higher than 10-fold was arbitrarily set as representing significant induction ([10](#_ENREF_10)).

**Analytical methods**

The column temperature program of the GC-FID was: 40°C hold for 2 min, followed by an increase of 6°C min^−1^ to 100°C and hold for 2 min, followed by further increase at 10°C min^−1^ to 225°C and hold for 2 min. The program for benzene measurement was as described earlier ([11](#_ENREF_11)). The wavelength of the UV detector of the HPLCs was 210 nm. The mobile phases for the Thermo Scientific Accela HPLC System were 0.1% formic acid in water (eluent A) and 0.1% formic acid in acetonitrile (eluent B). The mobile phase for the ThermoFisher Scientific SpectraSYSTEM™ HPLC was 0.01 N H_2_SO_4_. Halogenated phenols and phenol were analyzed using a three-step gradient profile consisting of: i) 90% eluent A and 10% eluent B for 2 min, ii) 90—20% eluent A and 10−80% eluent B for 14 min and hold at 20% eluent A and 80% eluent B for 3 min, iii) followed by 20—90% eluent A and 80—10% eluent B for 1 min. The ions were analyzed using a three-step gradient profile consisting of 1 mM KOH for 1 min, 1—40 mM KOH for 14 min and hold at 40 mM KOH for 4 min, followed by 40−1 mM KOH for 4.5 min.

**Supplementary Figures**


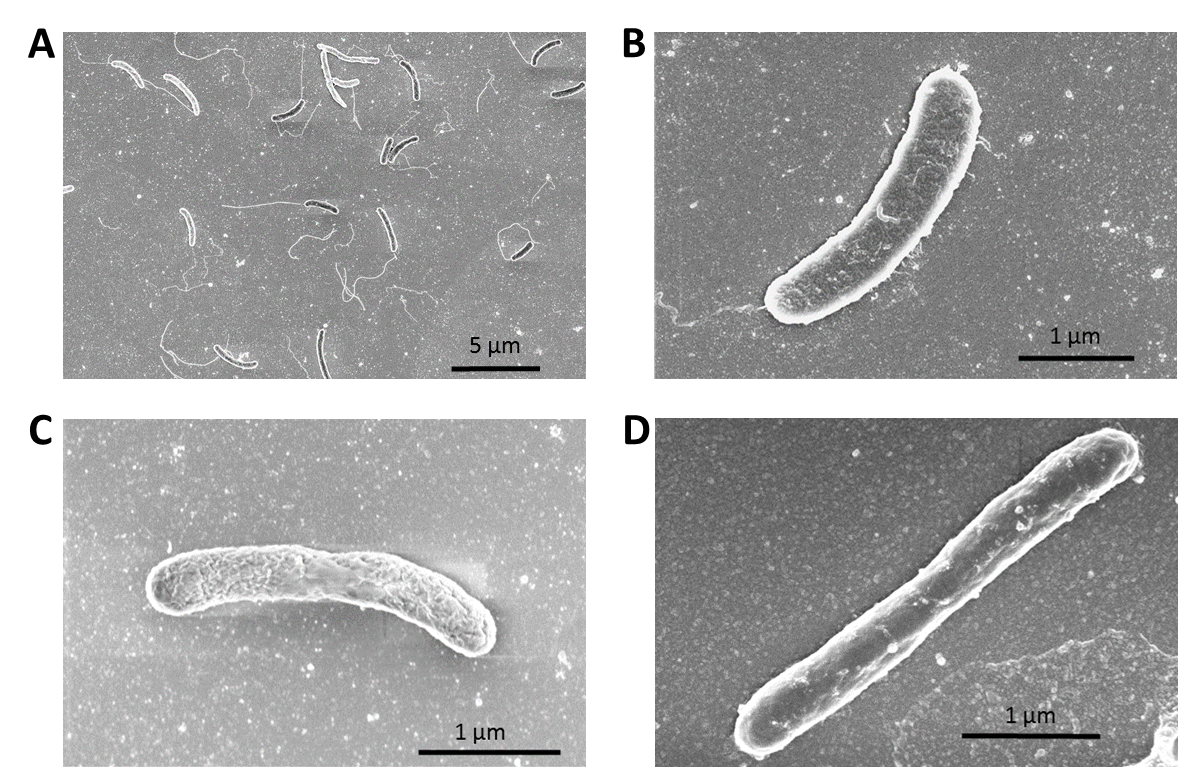


**Fig. S1** Scanning electron micrograph of *D. spongiiphila* DBB (A and B), *D. spongiiphila* AA1^T^ (C) and *D. butyratoxydans* MSL71^T^ (D).


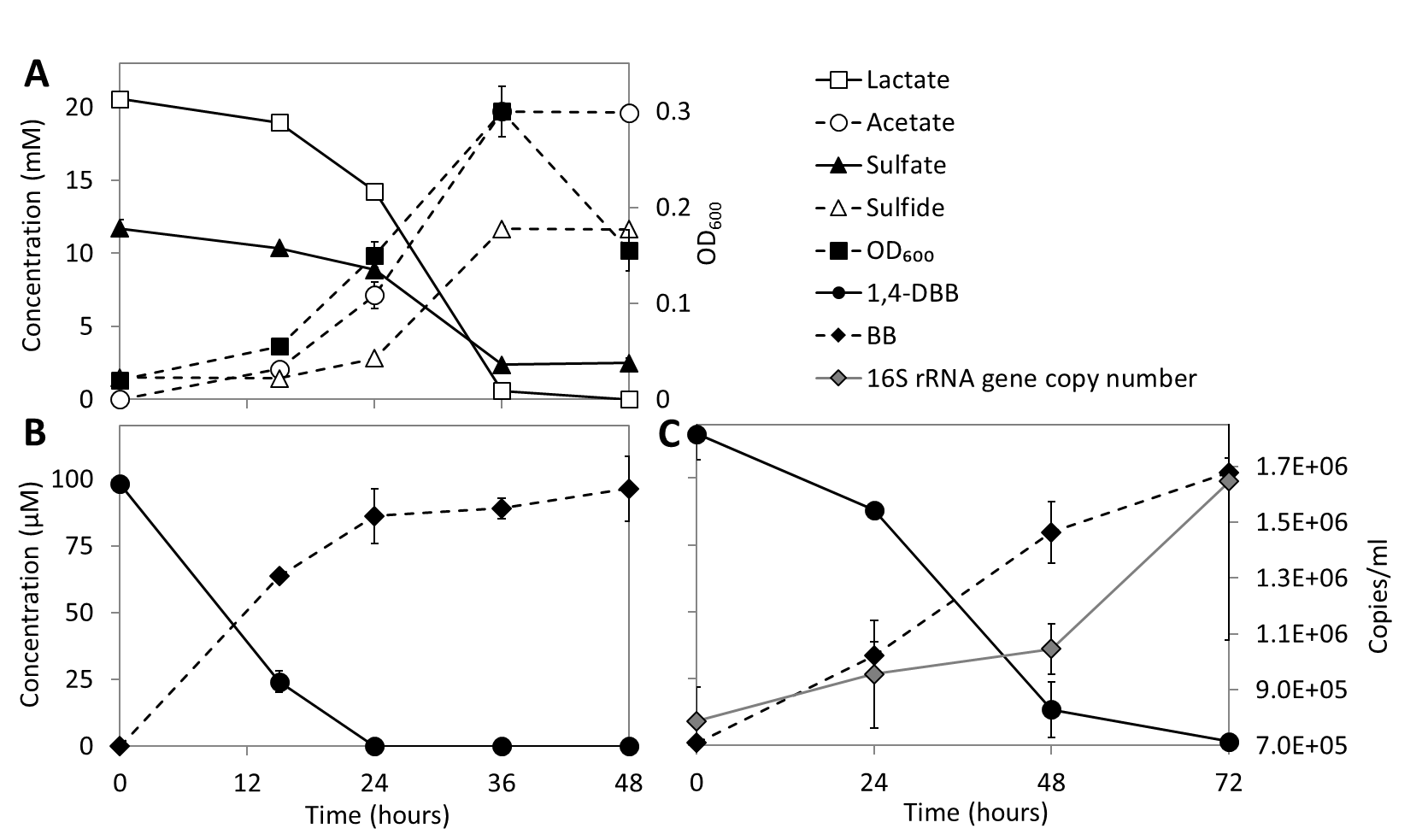


**Fig. S2** Concurrent 1,4-DBB debromination and sulfate reduction by strain DBB with lactate as the electron donor. Points and error bars represent the average and standard deviation of samples taken from duplicate cultures.


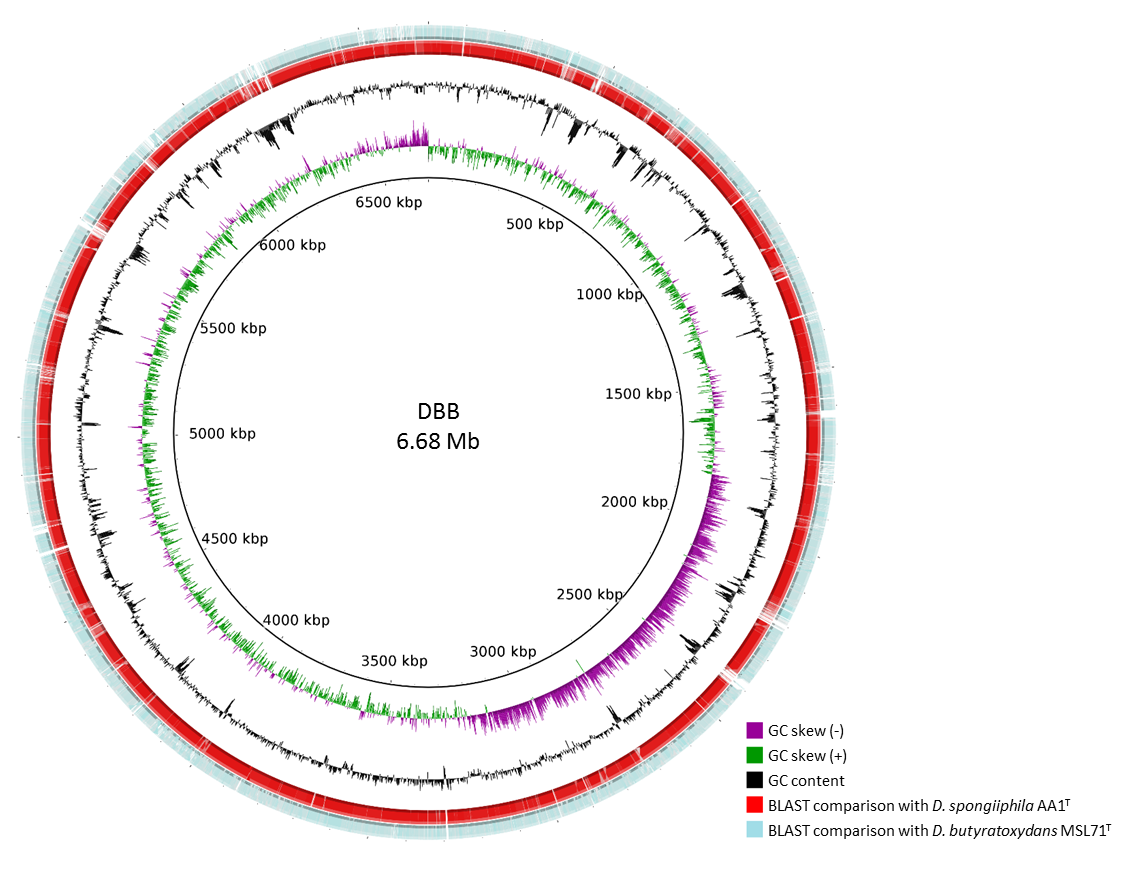


**Fig. S3** Circular representation of the genome sequence of *D. spongiiphila* DBB in comparison with the genomes of *D. spongiiphila* AA1^T^ and *D. butyratoxydans* MSL71^T^.


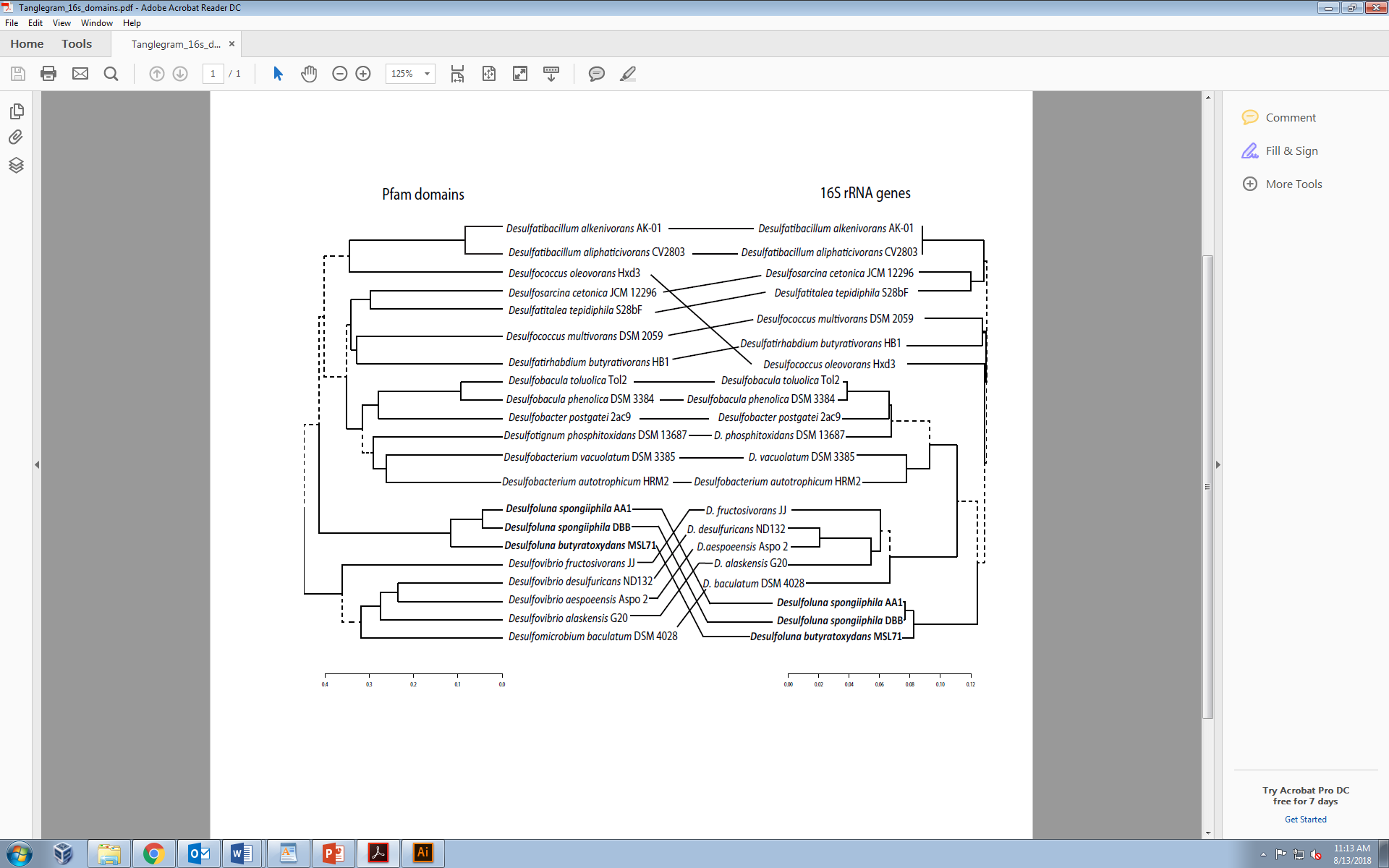


**Fig. S4** Phylogenetic tree based on 16S rRNA gene sequence and protein domain analyses. A comparison is included as horizontal lines between the two trees, showing the position of strain DBB relative to other strains belonging to the family *Desulfobacteraceae* as well as several *Desulfovibrio* strains. The “unique” nodes between the 16S rRNA gene- and domain-based tree are indicated with dashed lines. Genomes (Table S4) were selected based on the phylogenetic tree of the family *Desulfobacteraceae* ([12](#_ENREF_12)).


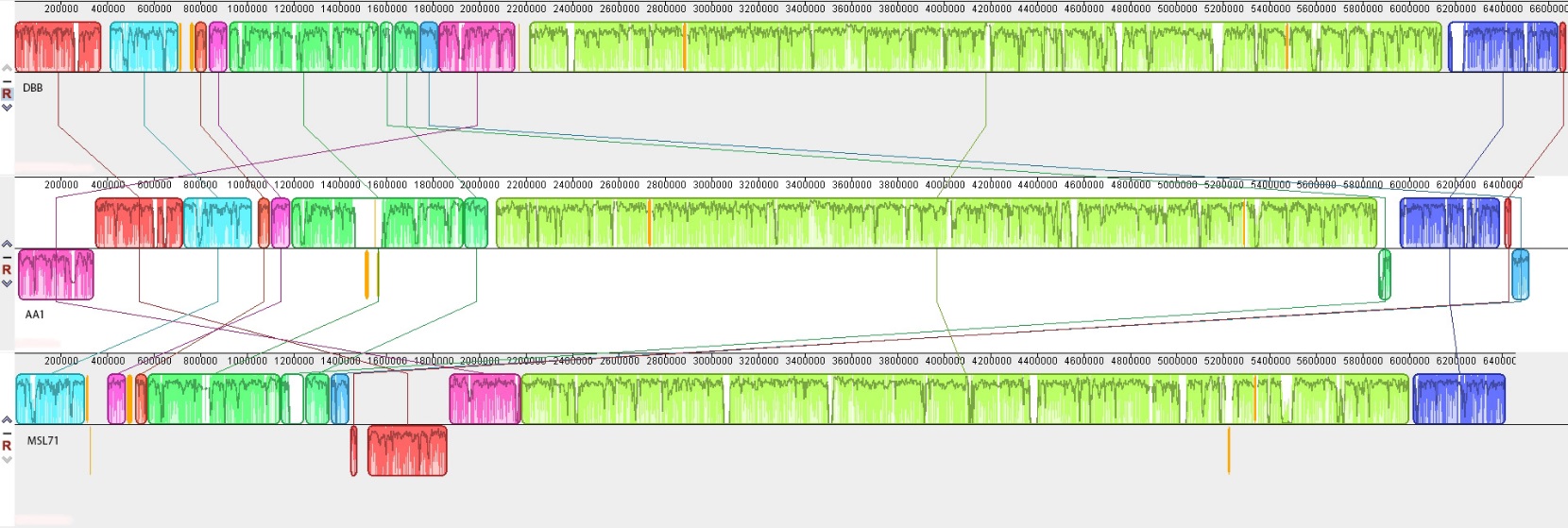


**Fig. S5** Whole genome alignment of *D. spongiiphila* DBB (Top), *D. spongiiphila* AA1^T^ (middle) and *D. butyratoxydans* MSL71^T^ (bottom). The genome of strain DBB was used as reference for global alignment using progressive MAUVE ([13](#_ENREF_13)). The locally collinear blocks (LCBs) that were identified in the genomes were outlined in frame. Conserved and highly related regions are coloured, and low-identity unique regions are in white (colorless). LCBs below the mid-line in *D. spongiiphila* AA1^T^ and *D. butyratoxydans* MSL71^T^ are inverted relative to *D. spongiiphila* DBB.


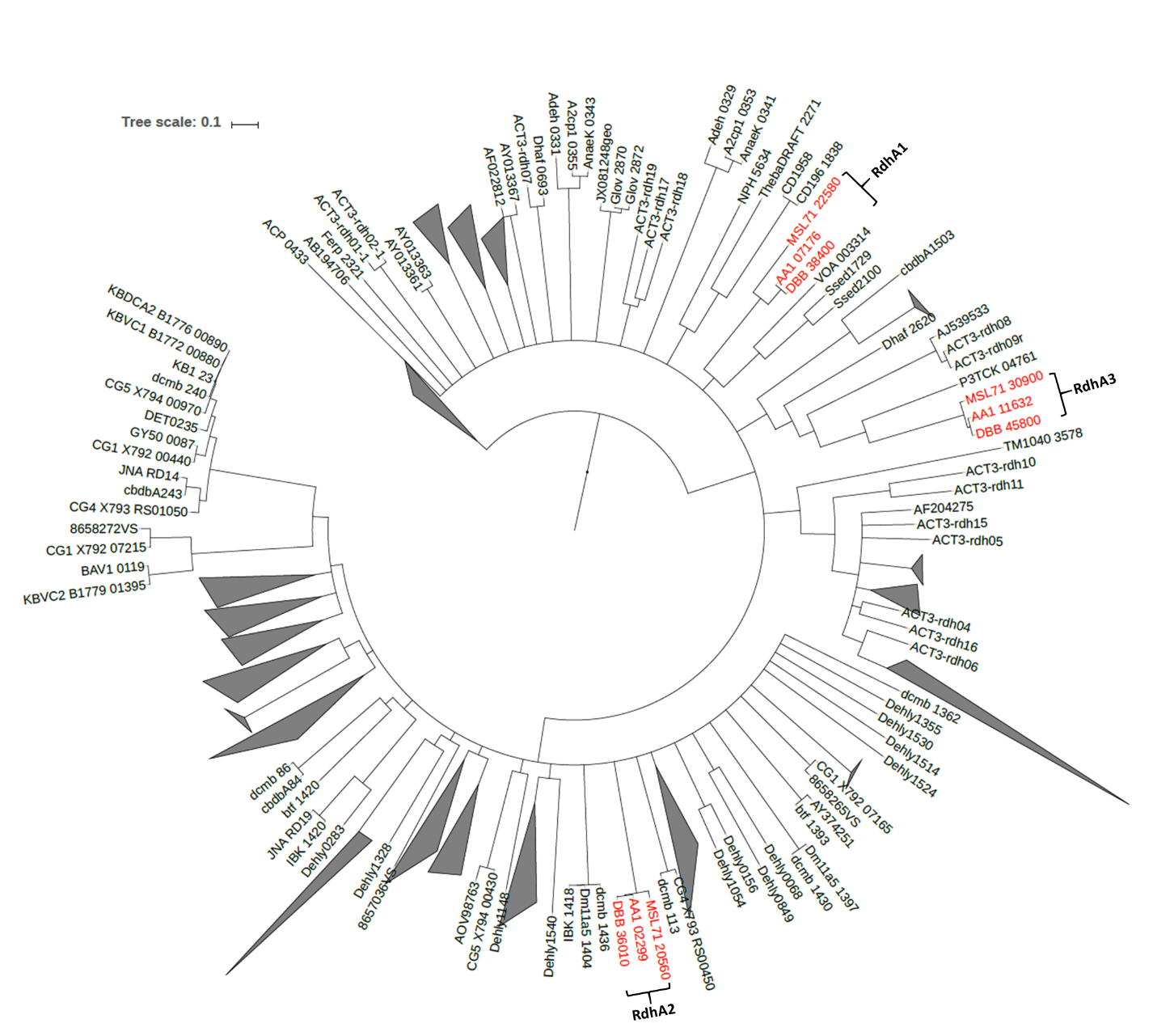


**Fig. S6** Phylogenetic analysis of the RdhAs of *Desulfoluna* strains and 548 RdhAs reported previously ([14](#_ENREF_14)). The RdhA sequences were obtained from the public link: <https://drive.google.com/drive/folders/0BwCzK8wzlz8ON1o2Z3FTbHFPYXc>. The multiple sequence alignment was processed using Geneious software with the MAFFT algorithm, and the phylogenetic tree was constructed using the same software with default settings. Further polishing of the phylogenetic tree was performed on the Interactive Tree of Life web browser (<http://itol.embl.de/>) ([15](#_ENREF_15)). The RdhAs of *Desulfoluna* strains are shown in red font.


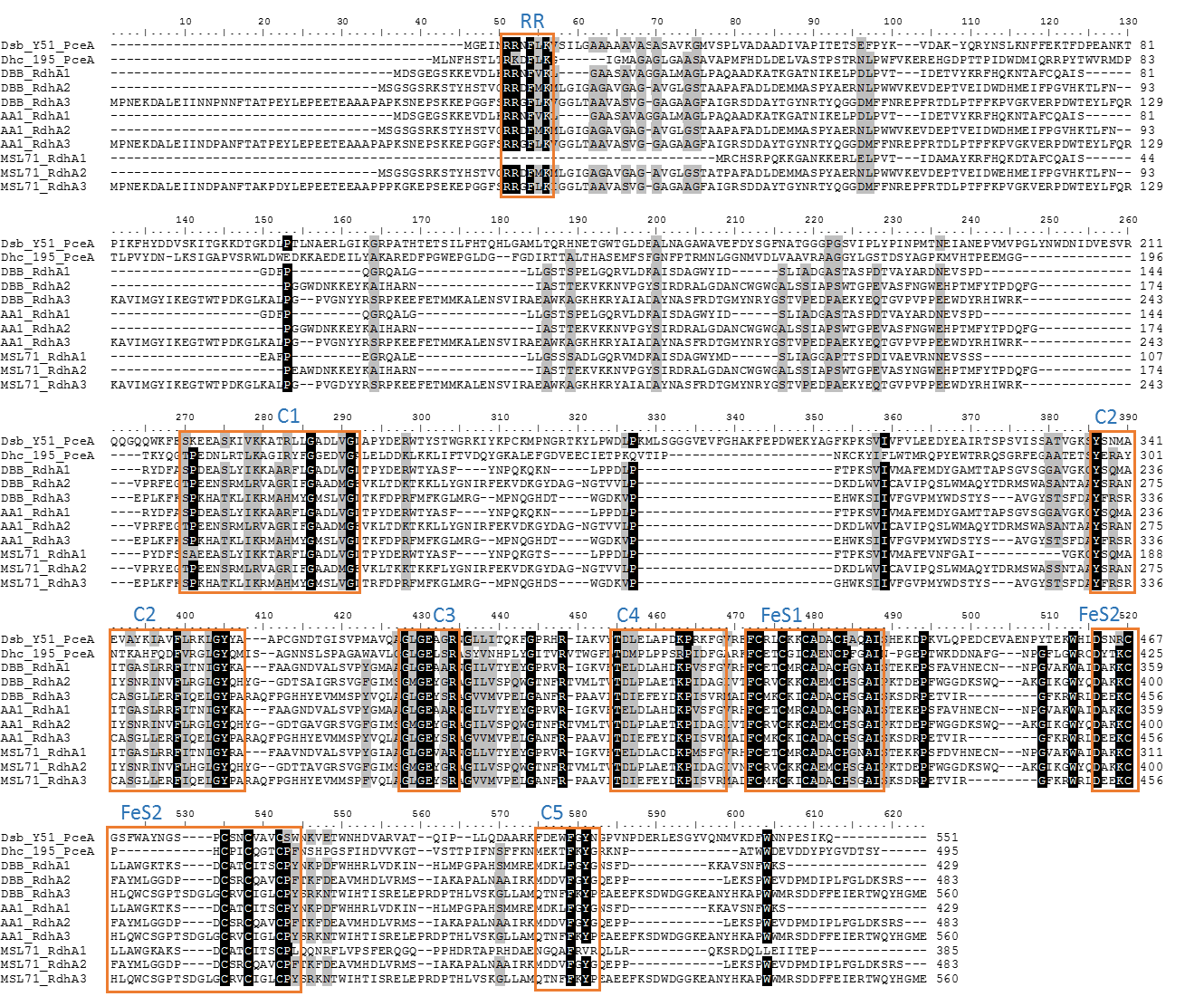


**Fig. S7** Multiple-sequence alignment of the RdhAs from *D. spongiiphila* DBB, *D. spongiiphila* AA1^T^ and *D. butyratoxydans* MSL71^T^ and two functionally characterized RdhAs from *Desulfitobacterium hafniense* Y51, and *Dehalococcoides mccartyi* strain 195. The conserved sequence motifs (RR, C1−C5, FeS1, and FeS2) are enclosed within orange boxes. The selected RdhAs (GenBank accession number or locus number) and corresponding bacteria are: Dsb_Y51_PceA: *D. hafniense* Y51, BAC00915. Dhc_195_PceA: *D. mccartyi* 195, Q3Z9N3. DBB_3755: *D. spongiiphila* DBB. DBB_3984: *D. spongiiphila* DBB. DBB_4749: *D. spongiiphila* DBB. AA1_02299: *D. spongiiphila* AA1^T^. AA1_07176: *D. spongiiphila* AA1^T^. DBB_11632: *D. spongiiphila* AA1^T^. MSL71_1800: *D. butyratoxydans* MSL71^T^. MSL71_2003: *D. butyratoxydans* MSL71^T^. MSL71_4258: *D. butyratoxydans* MSL71^T^. The conceived sequence motifs were defined according sequence analysis of RdhA from OHRB ([14](#_ENREF_14), [16](#_ENREF_16)).

**
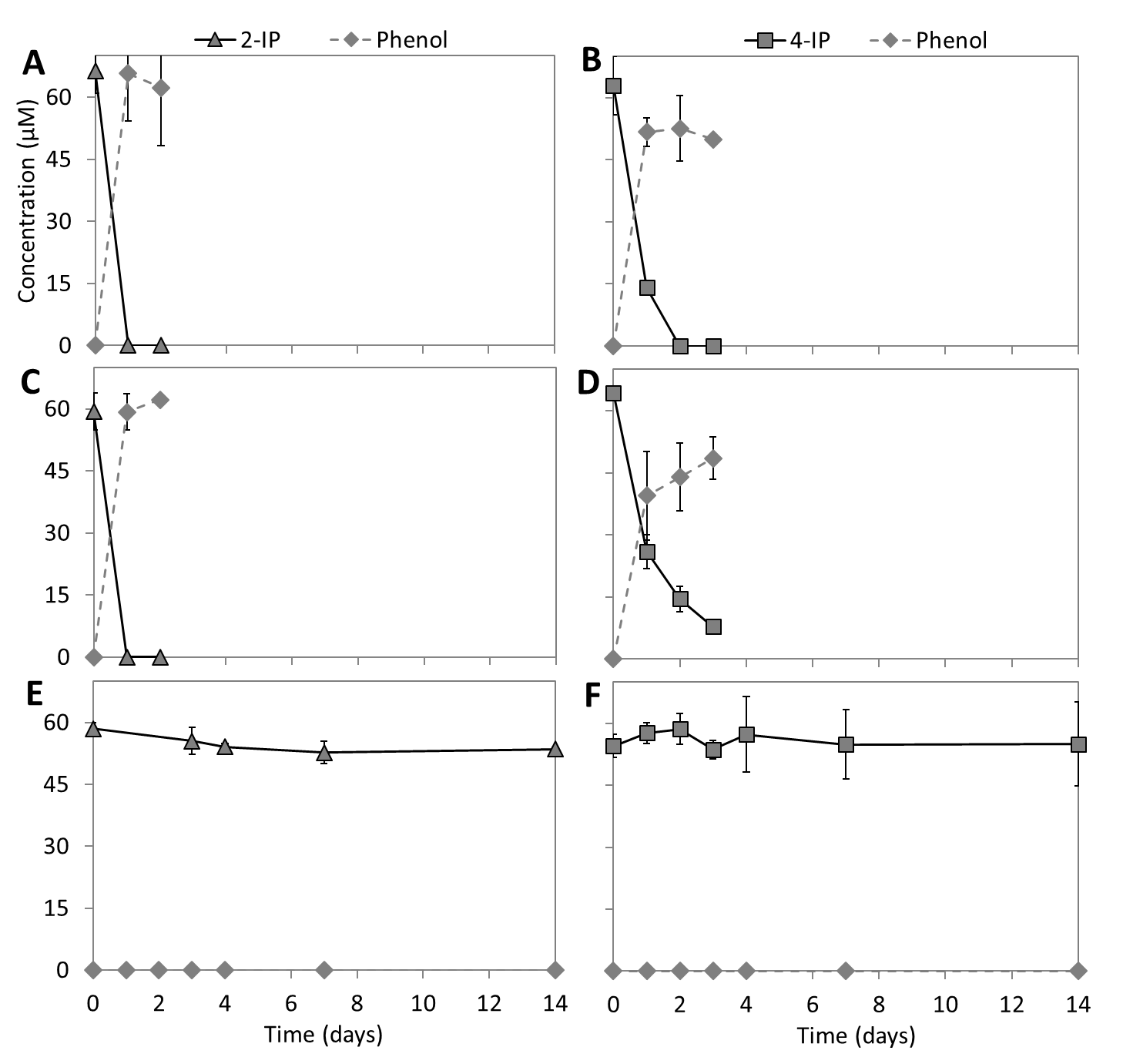
**
**Fig. S8** Deiodination of 2-IP and 4-IP by *D. spongiiphila* DBB (A, B), *D. spongiiphila* AA1^T^ (C, D) and *D. butyratoxydans* MSL71^T^ (E, F) with lactate (5 mM) as the electron donor. Points and error bars represent the average and standard deviation of samples taken from duplicate cultures.


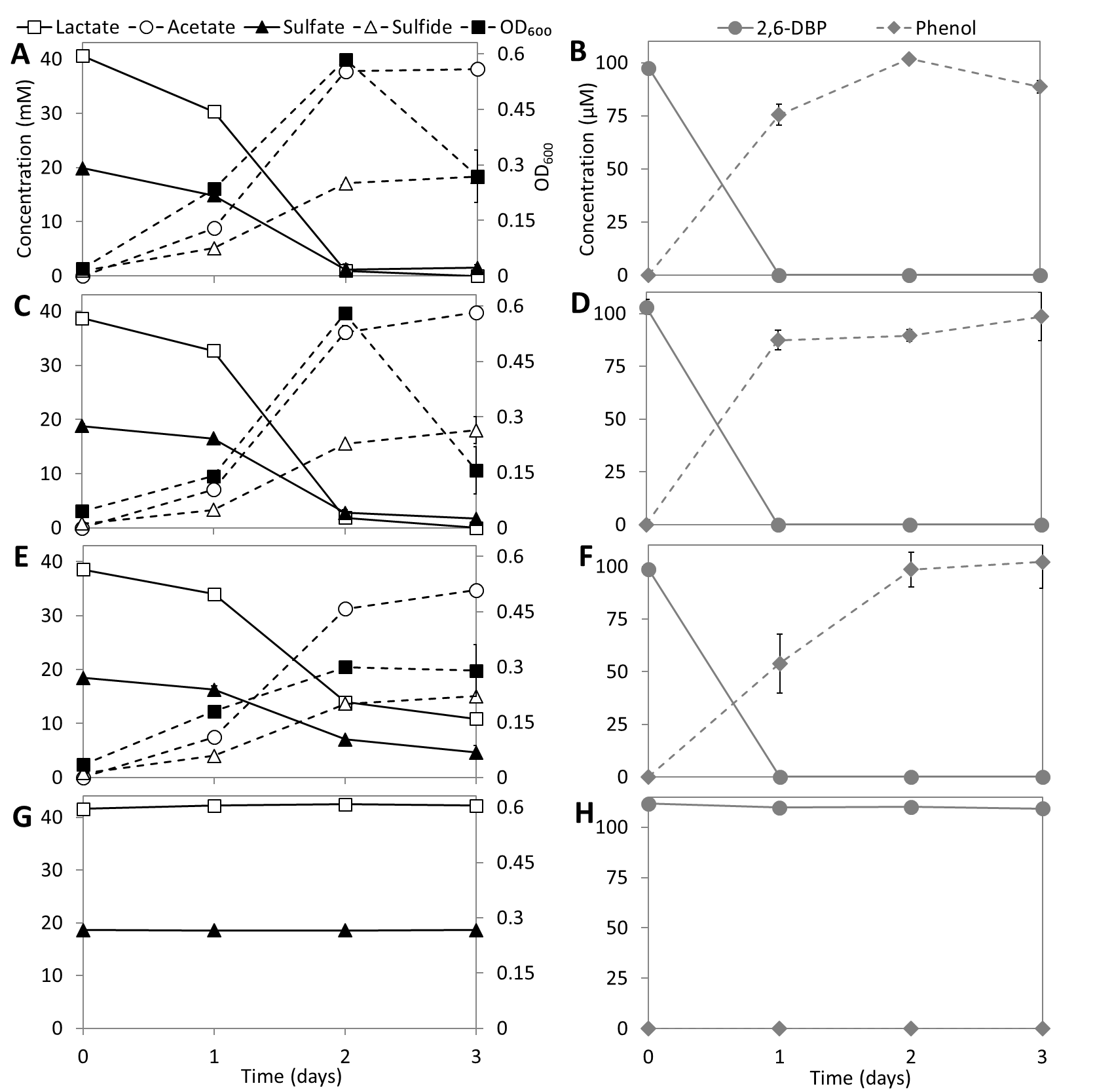


**Fig. S9** Concurrent debromination of 2,6-DBP (100 µM) and sulfate (20 mM) reduction by *D. spongiiphila* DBB (A, B), *D. spongiiphila* AA1^T^ (C, D) and *D. butyratoxydans* MSL71^T^ (E, F) with lactate (40 mM) as the electron donor. Negative controls (G, H) were prepared similarly except that they were not inoculated. Points and error bars represent the average and standard deviation of samples taken from duplicate cultures.


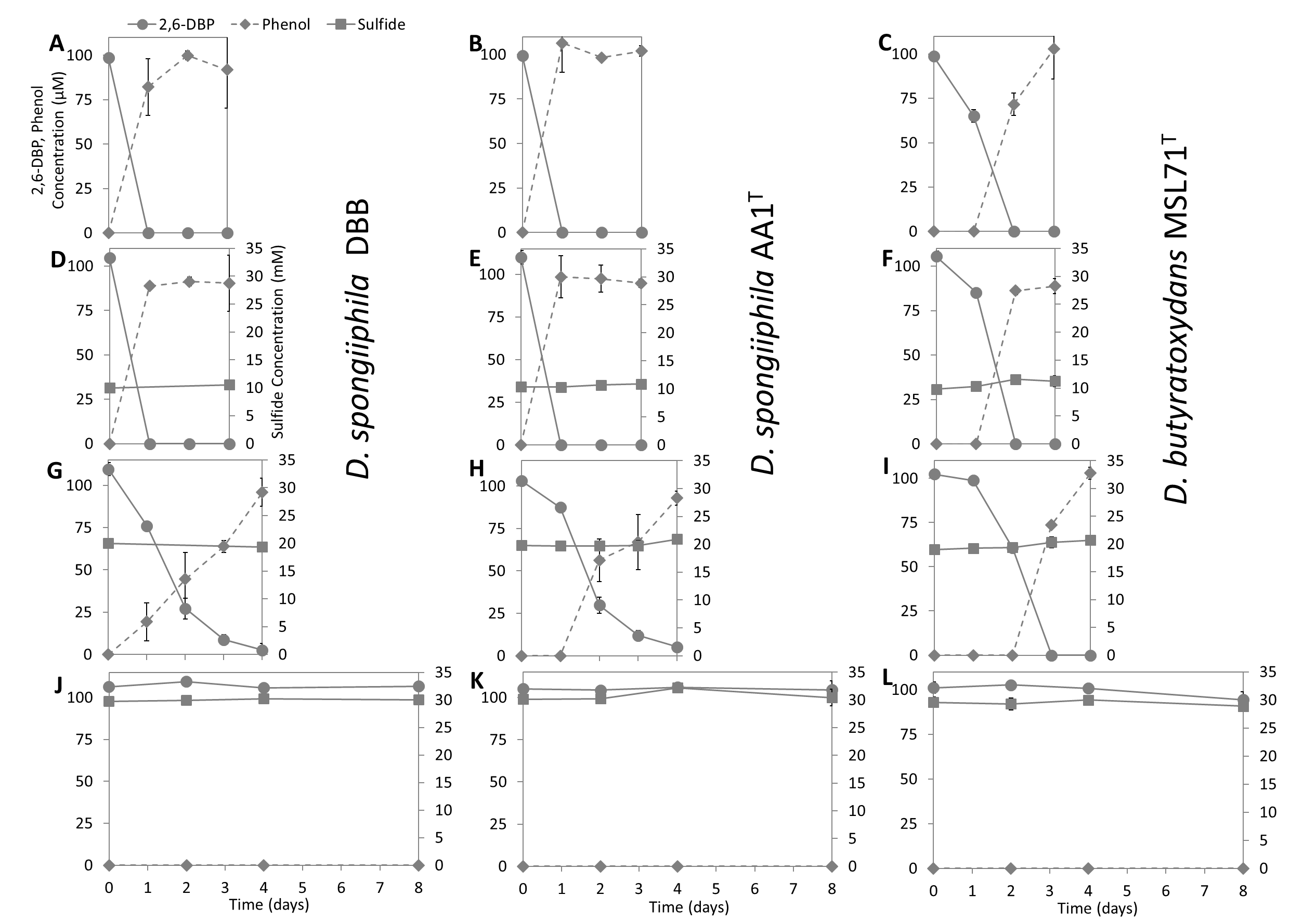


**Fig. S10** Debromination of 2,6-DBP by *D. spongiiphila* DBB (A, D, G, J), *D. spongiiphila* AA1^T^ (B, E, H, K) and *D. butyratoxydans* MSL71^T^ (C, F, I, L) in presence of 1 (A, B, C), 10 (D, E, F), 20 (G, H, I) and 30 mM (J, K, L) sulfide. Points and error bars represent the average and standard deviation of samples taken from duplicate cultures.


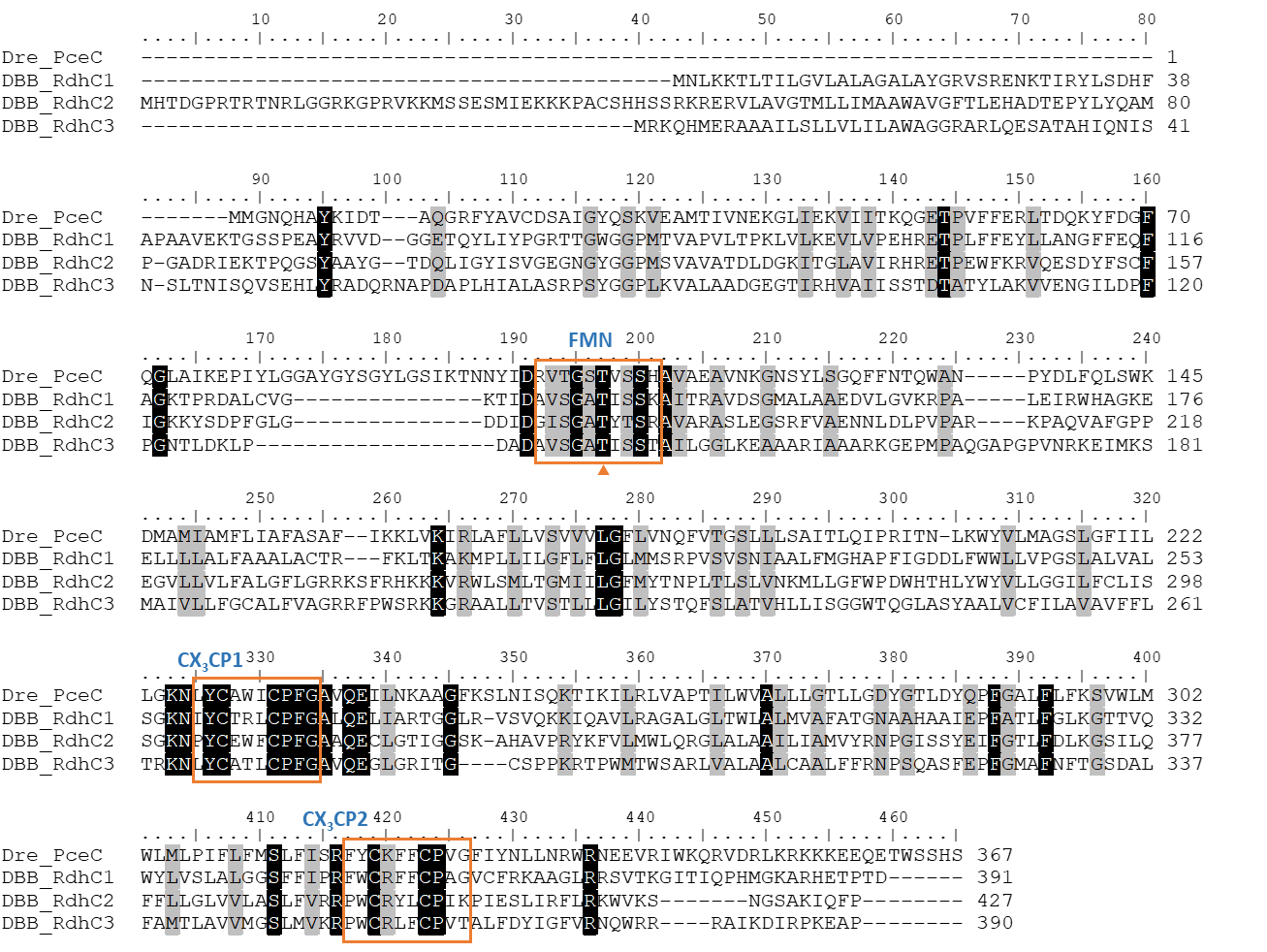


**Fig. S11** Multiple-sequence alignment of the RdhCs of *Desulfoluna spongiiphila* DBB and *Dehalobacter restrictus* (Dre) (GenBank accession number: CAG70347.1). The conserved FMN binding motifs and two CX_3_CP motifs are enclosed within orange boxes. The conserved threonine residue predicted to covalently bind to FMN is indicated with an orange triangle.


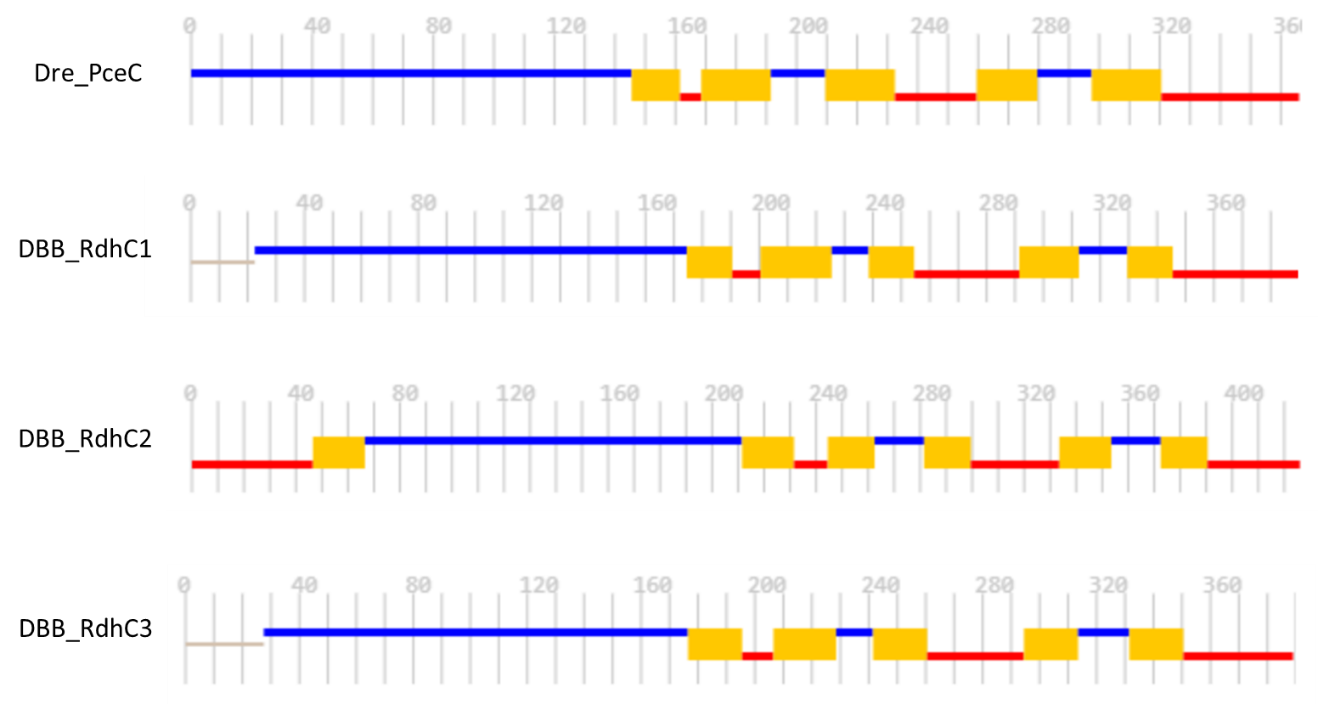


**Fig. S12** Topology analysis and comparison of the RdhCs in *D. spongiiphila* DBB and PceC of *Dehalobacter restrictus*. The topology was predicted using CCTOP ([17](#_ENREF_17)). Blue lines indicate outside/extra-cytosolic regions. Red lines indicate inside/cytosolic regions. Gray lines indicate regions where topology is not predicted. Yellow rectangles indicate transmembrane regions.


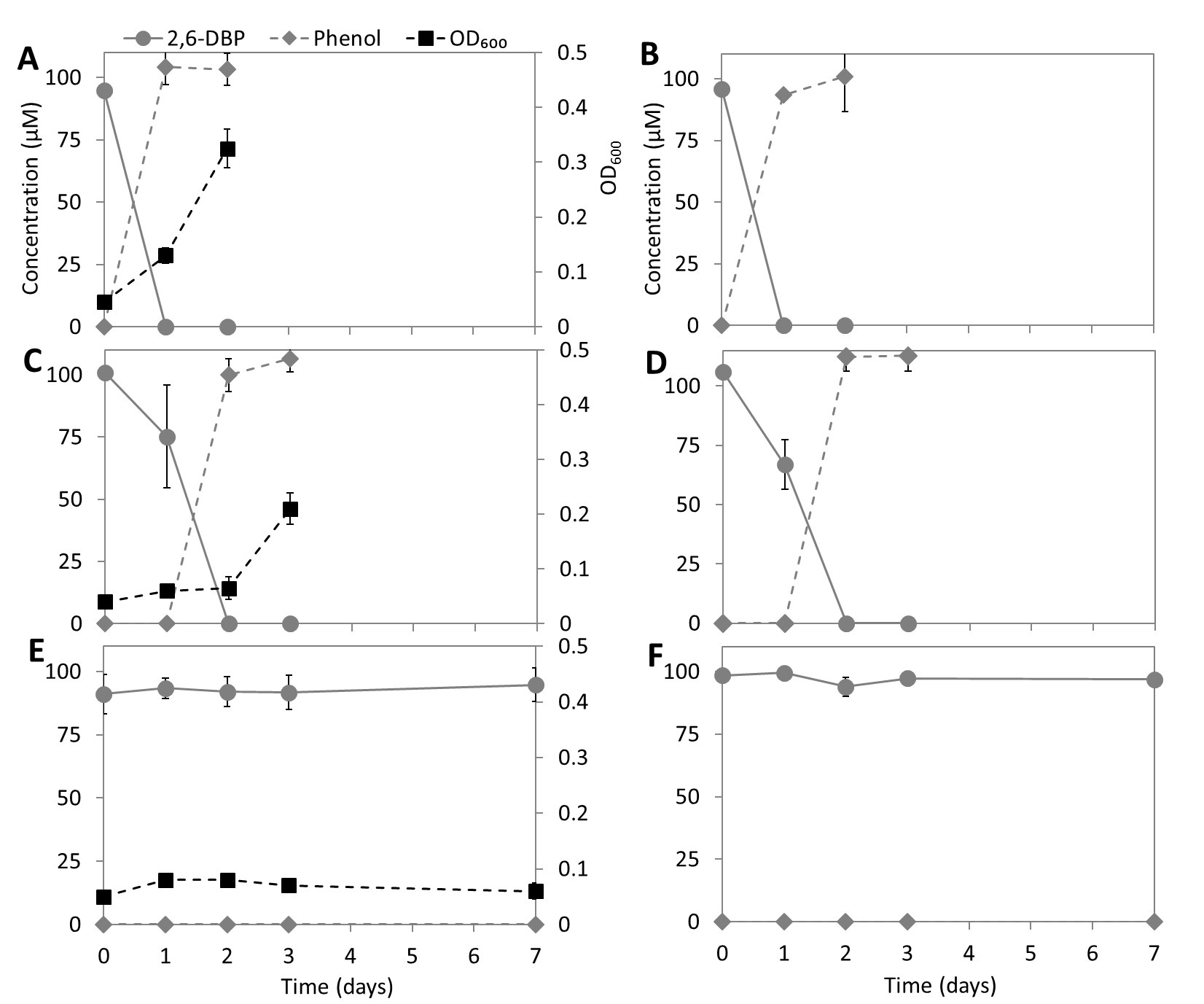


**Fig. S13** 2,6-DBP debromination by *D. spongiiphila* DBB grown in presence (A, C, E) or absence (B, D, F) of sulfate (20 mM) and initial oxygen concentration of 0% (A, B), 2% (C, D), 5% (E, F). Points and error bars represent the average and standard deviation of samples taken from duplicate cultures.

**Supplementary Tables**

Table S1. Primers used in this study

| Target | Name | Sequence (5´—3´)^a^ | Application | Reference |
| --- | --- | --- | --- | --- |
| Bacteria 16S rRNA | 27F-DegS | GTTYGATYMTGGCTCAG | Miseq | ([18](#_ENREF_18)) |
|  | 338R–I | GCWGCCTCCCGTAGGAGT | Miseq | ([19](#_ENREF_19)) |
|  | 338R–II | GCWGCCACCCGTAGGTGT |  |  |
|  | Unitag1 | GAGCCGTAGCCAGTCTGC | Miseq | ([20](#_ENREF_20)) |
|  | Unitag2 | GCCGTGACCGTGACATCG |  |  |
| Bacteria 16S rRNA | Eub341F | CCTACGGGAGGCAGCAG | RT-qPCR | ([21](#_ENREF_21)) |
|  | Eub534R | ATTACCGCGGCTGCTGGC |  |  |
| *rdhA1* ^b^ | Rdh1F | ACCGCTACGATTTTGCATCC | RT-qPCR | This study |
|  | Rdh1R | CCATCTCAAAGGCCATGACG |  |  |
| *rdhA*2 ^b^ | Rdh2F | CGTTATTCCGCAGTCGTTGT | RT-qPCR | This study |
|  | Rdh2R | CACTGGGGACTGACAAGGAT |  |  |
| *rdhA*3 ^b^ | Rdh3F | TGGCCGACTTTTGCATGAAA | RT-qPCR | This study |
|  | Rhd3R | AGGTGTTCTTGCGGGAGTAA |  |  |

^a^ M = A or C; R = A or G; W = A or T; Y = C or T

^b^ *rdhA* genes of *D. spongiiphila* DBB

Table S2. Cellular fatty acid composition (%) of different *Desulfoluna* strains

| Fatty acid | *D. spongiiphila* DBB | *D. spongiiphila* AA1^T^ | *D. butyratoxydans* MSL71^T^ |
| --- | --- | --- | --- |
| C12:0 | 0.3 | 0.3 | 0.8 |
| C14:1ω7 | 0.9 | 0.3 | 1.1 |
| C14:1ω5 | 0.2 | 0.1 | 0.2 |
| C14:0 | 9.9 | 6.6 | 11.5 |
| C16:1ω9 | 1.4 | 1.1 | 1.5 |
| C16:1ω7c | 19.4 | 18.9 | 22.4 |
| C16:1ω7tr | 0.3 | 0.3 | 0.3 |
| C16:1ω5 | 0.6 | 0.8 | 0.7 |
| C16:0 | 28.1 | 28.4 | 21.7 |
| C18:1ω9 | 11.6 | 11.8 | 11.3 |
| C18:1ω7 | 18.6 | 22.9 | 19.7 |
| C18:0 | 2.2 | 1.1 | 1.7 |
| β-OH-C12 | 0.4 | 0.5 | 0.4 |
| β-OH-C14 | 6.2 | 7.0 | 6.7 |

Table S3. General genome features the two *Desulfoluna* strains sequenced in this study

|  | DBB | MSL71^T^ |
| --- | --- | --- |
| Total read | 38,380,710^a^/  89,972^b^ | 31,677,790^a^ |
| Total bases | 9,595,177,500^a^/  379,389,288^b^ | 7,919,447,500^a^ |
| Completeness (%) | 99.35 | 100 |
| Contamination (%) | 1.61 | 1.29 |
| Total tRNA genes | 83 | 81 |
| Total tmRNA genes | 1 | 1 |

^a^ According to Illumina sequencing data

^b^ According to Pacbio sequencing data Table S4. Selected genomes and their general features that were used for 16S rRNA gene and protein domain based phylogenetic analyses

| Strain ^a^ | Genome size (Mb) | GC (%) | Proteins | GenBank accession number |
| --- | --- | --- | --- | --- |
| *Desulfoluna spongiiphila* DBB | 6.68 | 57.1 | 5301 | GCA_902498735 |
| *Desulfoluna butyratoxydans* MSL71^T^ | 6.05 | 57.9 | 4186 | GCA_900699765 |
| *Desulfoluna spongiiphila* AA1^T^ | 6.53 | 57.2 | 5203 | NZ_FMUX01000001.1 |
| *Desulfatibacillum aliphaticivorans* CV2803 | 6.47 | 54.4 | 5264 | NZ_AUCT00000000.1 |
| *Desulfovibrio fructosivorans* JJ | 4.67 | 63.9 | 4046 | NZ_AECZ01000001.1 |
| *Desulfatibacillum alkenivorans* AK-01 | 6.49 | 54.7 | 5277 | NC_011768.1 |
| *Desulfatirhabdium butyrativorans* HB1 | 4.48 | 54.9 | 3852 | NZ_KE386985.1 |
| *Desulfatitalea tepidiphila* S28bF | 5.61 | 56.7 | 4858 | NZ_BCAG01000003.1 |
| *Desulfobacter postgatei* 2ac9 | 3.97 | 47.2 | 3845 | NZ_CM001488.1 |
| *Desulfobacterium autotrophicum* HRM2 | 5.65 | 48.7 | 4835 | NC_012108.1 |
| *Desulfobacterium vacuolatum* DSM 3385 | 5.03 | 46.5 | 4050 | NZ_FWXY01000001.1 |
| *Desulfobacula phenolica* DSM 3384 | 4.87 | 41.4 | 4181 | NZ_FNLL01000001.1 |
| *Desulfobacula toluolica* Tol2 | 5.19 | 41.4 | 4545 | NC_018645.1 |
| *Desulfococcus multivorans* DSM 2059 | 4.42 | 56.8 | 3783 | NZ_CP015381.1 |
| *Desulfococcus oleovorans* Hxd3 | 3.94 | 56.2 | 3325 | NC_009943.1 |
| *Desulfomicrobium baculatum* DSM 4028 | 3.94 | 58.6 | 3395 | NC_013173.1 |
| *Desulfosarcina cetonica* JCM 12296 | 7.09 | 55.7 | 5582 | NZ_BBCC01000001.1 |
| *Desulfotignum phosphitoxidans* DSM 13687 | 4.99 | 51.3 | 4556 | NZ_APJX01000001.1 |
| *Desulfovibrio aespoeensis* Aspo 2 | 3.62 | 62.6 | 3257 | NC_014844.1 |
| *Desulfovibrio alaskensis* G20 | 3.64 | 57.9 | 3270 | NC_007519.1 |
| *Desulfovibrio desulfuricans* ND132 | 3.85 | 65.2 | 3423 | NC_016803.1 |

^a^ Genome information were obtained from GenBank under their respective accession numbers, except *D. spongiiphila* DBB and *D. butyratoxydans* MSL71^T^

Table S5. Abundance of the proteins involved in lactate, sulfate and 1,4-DBB metabolism in cells of strain DBB grown in lactate plus sulfate (LS condition) and lactate plus sulfate plus 1,4-DBB (LSD condition). Values of the corresponding fraction with higher abundance are given; except for RdhA both membrane and soluble fraction values are given

|  | Locus tag | LS1 Area_log2_ | | LS2 Area_log2_ | LS3 Area_log2_ | LSD1 Area_log2_ | LSD2 Area_log2_ | LSD3 Area_log2_ | log2 fold-change | *p*-value |
| --- | --- | --- | --- | --- | --- | --- | --- | --- | --- | --- |
| **Putative proteins involved in lactate metabolism** | | | | | | | | | | |
| Lactate permease | 24890 | 22.96 | 23.17 | | 24.12 | 23.64 | 23.48 | 24.38 | 0.42 | 0.40 |
| LdhA-1 | 24880 | 28.72 | 28.66 | | 29.09 | 29.09 | 29.01 | 28.59 | 0.08 | 0.73 |
| LdhA-2 | 24970 | 28.03 | 28.08 | | 28.37 | 27.89 | 27.85 | 27.88 | -0.28 | 0.05 |
| LdhB-1 | 24870 | 25.39 | 25.97 | | 26.61 | 26.50 | 25.84 | 25.93 | 0.10 | 0.81 |
| LdhB-2 | 24960 | 26.35 | 26.69 | | 29.20 | 26.84 | 25.89 | 26.12 | -1.13 | 0.29 |
| Por-1 | 310 | 31.58 | 31.55 | | 31.62 | 30.70 | 30.64 | 30.79 | -0.87 | 0.00 |
| Por-2 | 24940 | 32.20 | 32.22 | | 32.18 | 31.20 | 31.05 | 31.25 | -1.03 | 0.00 |
| Pta | 9370 | 27.45 | 27.31 | | 27.91 | 27.97 | 27.56 | 27.57 | 0.15 | 0.55 |
| Ack | 9360 | 28.26 | 28.72 | | 28.92 | 29.10 | 28.35 | 28.27 | -0.06 | 0.86 |
| **Putative proteins involved in sulfate metabolism** | | | | | | | | | | |
| Sulfate permease | 22290 | 21.25 | 21.21 | | 22.95 | 22.35 | 22.14 | 22.49 | 0.52 | 0.42 |
| Sat | 23930 | 29.71 | 30.25 | | 29.73 | 30.04 | 30.03 | 29.50 | -0.04 | 0.88 |
| ApsBA | 23880 | 26.22 | 25.94 | | 26.11 | 26.33 | 26.34 | 25.92 | 0.11 | 0.54 |
|  | 23890 | 29.21 | 28.63 | | 29.02 | 29.22 | 28.85 | 28.57 | -0.08 | 0.78 |
| QmoABC | 23900 | 23.92 | 23.45 | | 24.16 | 24.36 | 23.64 | 24.40 | 0.29 | 0.42 |
|  | 23910 | 24.18 | 23.44 | | 24.48 | 24.88 | 24.32 | 24.36 | 0.48 | 0.25 |
|  | 23920 | 26.10 | 26.30 | | 26.72 | 26.22 | 26.24 | 26.34 | -0.11 | 0.59 |
| DsrC | 370 | 31.27 | 31.08 | | 31.57 | 31.58 | 30.91 | 31.38 | -0.02 | 0.95 |
| DsrABD | 25620 | 32.60 | 32.22 | | 32.75 | 32.50 | 32.82 | 32.47 | 0.08 | 0.71 |
|  | 25630 | 32.66 | 32.45 | | 32.50 | 32.32 | 32.18 | 32.31 | -0.26 | 0.03 |
|  | 25640 | 29.06 | 28.67 | | 29.24 | 29.11 | 29.04 | 28.98 | 0.06 | 0.77 |
| DsrMKJOP | 27290 | 22.84 | 22.29 | | 22.87 | 22.46 | 21.95 | 23.37 | -0.08 | 0.87 |
|  | 27300 | 22.72 | 23.43 | | 24.32 | 24.46 | 23.24 | 22.96 | 0.07 | 0.92 |
|  | 27310 | ND | ND | | ND | ND | ND | ND | - | - |
|  | 27320 | 22.90 | 22.59 | | 22.90 | 22.28 | 19.51 | 22.20 | -1.46 | 0.18 |
|  | 27330 | 22.80 | 22.52 | | 23.81 | 22.72 | 23.09 | 23.97 | 0.22 | 0.70 |
| **Putative electron transport proteins** | | | | | | | | | | |
| Flavodoxin | 37290 | 33.29 | 33.59 | | 31.86 | 32.73 | 33.16 | 33.91 | 0.35 | 0.61 |
| QrcABCD | 34140 | ND | ND | | ND | ND | ND | ND | - | - |
|  | 34150 | 22.42 | 21.33 | | 22.31 | 20.66 | 21.51 | 21.22 | -0.89 | 0.11 |
|  | 34160 | 22.18 | 22.28 | | 22.82 | 22.10 | 22.60 | 22.56 | -0.01 | 0.97 |
|  | 34170 | ND | ND | | ND | ND | ND | ND | - | - |
| Reductive dehalogenase | | | | | | | | | | |
| RdhA1 | 38400 | ND | ND | | ND | 26.62 | 25.99 | 25.39 | - | - |
| RdhA1 | 38400 | ND | ND | | ND | 24.36 | 23.92 | 24.22 | - | - |
| Putative corrinoid biosynthesis proteins | | | | | | | | | | |
| GltX | 24080 | 25.00 | 24.89 | | 24.99 | 24.83 | 24.45 | 24.66 | -0.31 | 0.05 |
| HemL | 7500 | 26.66 | 27.12 | | 26.78 | 26.39 | 26.04 | 24.88 | -1.08 | 0.08 |
| HemB | 44050 | 26.25 | 26.89 | | 26.29 | 25.94 | 25.78 | 26.45 | -0.42 | 0.22 |
| HemC | 18940 | 27.66 | 27.70 | | 27.57 | 27.16 | 27.31 | 27.64 | -0.27 | 0.14 |
| HemD | 18950 | 27.94 | 27.84 | | 27.70 | 27.71 | 27.83 | 26.97 | -0.32 | 0.31 |
| CysG | 26600 | 24.85 | 25.24 | | 25.21 | 25.04 | 25.02 | 25.05 | -0.06 | 0.63 |
| CbiK | 3730 | 22.33 | 23.47 | | 22.80 | 21.70 | 22.66 | 22.96 | -0.43 | 0.44 |
| CobH/CbiC | 3780 | ND | 22.07 | | ND | 22.57 | 21.98 | 21.52 | - | - |
| CbiL | 3790 | 24.06 | 24.62 | | 23.45 | 24.55 | 23.59 | 24.36 | 0.12 | 0.79 |
| CbiH | 3850 | 23.81 | 24.54 | | 24.96 | 25.56 | 25.70 | 26.10 | 1.35 | 0.02 |
| CobU | 3880 | 24.33 | 22.29 | | ND | 24.24 | ND | ND | - | - |

The proteome raw data can be found in supplementary dataset 1–3

The full name of each protein can be found in Figure 5 and Table S6, subunits of protein complexes (e.g. MKJOP) are given in the order of the corresponding locus tags.

ND: not detected, dark grey: membrane fraction, light grey: soluble fraction, light blue: imputed value

Table S6. Corrinoid biosynthesis pathways and corresponding genes and functions in *Desulfoluna* strains. The arrows indicate transformation of each compounds to the subsequent product. The first six genes are responsible for heme biosynthesis

| Biosynthetic pathway | Gene ^a^ | Gene locus number (DBB) ^b^ | Gene locus number (AA1^T^) ^c^ | Gene locus number (MSL71^T^) ^b^ | Function in corrinoid biosynthesis |
| --- | --- | --- | --- | --- | --- |
| Glutamate |  |  |  |  |  |
| ↓ | *gltX* ^d^ | **24080** | 11166 | 15970 | Glutaminyl-trna synthetase |
| ↓ | *hemA* ^d^ | 26620 | 12922 | 13190 | Glutamyl-trna reductase |
| ↓ | *hemL* ^d^ | **7500** | 10673 | 46530 | Glutamate-1-semialdehyde 2,1-aminomutase |
| ↓ | *hemB* ^d^ | **44050** | 13043 | 37790 | Porphobilinogen synthase |
| ↓ | *hemC* ^d^ | **18940** | 103136 | 7120 | Hydroxymethylbilane synthase |
| ↓ | *hemD* ^d^ | **18950** | 103137 | 7130 | Uroporphyrinogen-III synthase |
| Uroporpyhrinogen III |  |  |  |  |  |
| ↓ | *cysG* | **26600** | 12920 | 13210 | Uroporphyrin-III C-methyltransferase |
| Precorrin-2 |  |  |  |  |  |
| ↓ | *cbiK* | **3730** | 12810 | 49290 | Sirohydrochlorin cobaltochelatase |
| Co(II)- precorrin-2 |  |  |  |  |  |
| ↓ | *cbiL* | **3790** | 12816 | 49350 | Precorrin-2/cobalt-factor-2 C20-methyltransferase |
| Co(II)- precorrin-3 |  |  |  |  |  |
| ↓ | *cbiH* | **3850** | 12822 | 49410 | Precorrin-3B C17-methyltransferase |
| Co(II)-precorrin-4 |  |  |  |  |  |
| ↓ | *cbiF* | 3830 | 12820 | 49390 | Precorrin-4/cobalt-precorrin-4 C11-methyltransferase |
| Co(II)-precorrin-5A |  |  |  |  |  |
| ↓ | *cbiG* | 3840 | 12821 | 49400 | Cobalt-precorrin 5A hydrolase |
| Co(II)-precorrin-5B |  |  |  |  |  |
| ↓ | *cbiD* | 3810 | 12818 | 49370 | Cobalt-precorrin-5B (C1)-methyltransferase |
| Co(II)-precorrin-6A |  |  |  |  |  |
| ↓ | *cbiJ* ^e^ |  |  |  | Precorrin-6A/cobalt-precorrin-6A reductase |
| Co(II)-precorrin-6B |  |  |  |  |  |
| ↓ | *cbiET* | 3820 | 12819 | 49380 | Cobalt-precorrin-6B (C15)-methyltransferase |
| Co(II)-precorrin-7,8 |  |  |  |  |  |
| ↓ | *cbiC* | **3780** | 12815 | 49340 | Precorrin-8X/cobalt-precorrin-8 methylmutase |
| Cobyrinic acid |  |  |  |  |  |
| ↓ | *cbiA* | 3770 | 12814 | 49330 | Cobyrinic acid *a,c*-diamide synthase |
| Cob(II)yrinic acid *a,c*-diamide |  |  |  |  |  |
| ↓ |  |  |  |  |  |
| Cob(I)yrinic acid *a,c*-diamide |  |  |  |  |  |
| ↓ | *cobA* | 3860 | 12823 | 49420 | Cob(I)alamin adenosyltransferase |
| Ado-cob(I)yrinic acid *a,c*-diamide |  |  |  |  |  |
| ↓ | *cbiP* | 3870 | 12824 | 49430 | Adenosylcobyric acid synthase |
| Adenosyl cobyrinate *a,c*-hexaamide |  |  |  |  |  |
| ↓ | *cbiB* | 3920 | 12829 | 49480 | Adenosylcobinamide-phosphate synthase |
| Ado-cobinamide |  |  |  |  |  |
| ↓ | *cobU* | **3880** | 12825 | 49440 | Adenosylcobinamide-phosphate guanylyltransferase |
| Ado-cobinamide-GDP |  |  |  |  |  |
| ↓ | *cobS* | 3890 | 12826 | 49450 | Cobalamin synthase |
| Cobalamin |  |  |  |  |  |

^a^ Gene nomenclature for the anaerobic corrinoid biosynthesis pathway was as previously published ([22](#_ENREF_22))

^b^ Gene Locus numbers are according the genome sequences of strains DBB and MSL71^T^ sequenced in this study. The numbers shown in bold are proteins detected in the proteome of strain DBB

^c^ Gene Locus numbers are according the genome sequence of stain AA1^T^ in GenBank

^d^ Genes responsible for heme biosynthesis

^e^ Genes that were not found in the *Desulfoluna* genomes

Table S7. Sulfur metabolism pathways and corresponding genes in *Desulfoluna* strains. The arrows indicate transformation of each compounds to the subsequent product

| Metabolic pathway | Enzyme | DBB ^a^ | AA1^T b^ | MSL71^T a^ |
| --- | --- | --- | --- | --- |
|  | Sulfate permease | 13340, **22290**, 24700, 47100 | 12364, 11742, 111127, 104190 | 27680, 17610, 15410, 29700 |
| Tetrathionate |  |  |  |  |
| ↓ | Tetrathionate reductase | 32070, 32080 | 11084, 11085 | ND |
| Thiosulfate |  |  |  |  |
| ↓ | Molybdopterin oxidoreductase  or  Rhodanese-like protein^c^ | 8600—8620    9670, 10640 | 106177—106179  10968, 10571 | 41260—41280  51880, 25130 |
| Sulfite |  |  |  |  |
| Sulfate |  |  |  |  |
| ↓ | Sulfate adenylyltransferase | **23930** | 11149 | 16120 |
| Adenylyl sulfate (APS) |  |  |  |  |
| ↓ | APS reductase α subunit | **23890** | 11145 | 16160 |
|  | APS reductase β subunit | **23880** | 11144 | 16170 |
| Sulfite |  |  |  |  |
| ↓ | Dissimilatory sulfite reductase α subunit | **25620** | 12530 | 14160 |
|  | Dissimilatory sulfite reductase β subunit | **25630** | 12529 | 14150 |
|  | Dissimilatory sulfite reductase D | **25640** | 12528 | 14140 |
| Sulfide |  |  |  |  |

^a^ Locus tag numbers are according to the genome of strains DBB and MSL71^T^ sequenced in this study Numbers shown in bold are proteins detected in the proteome of strain DBB

^b^ Locus tag numbers are according to the genome of stain AA1^T^ (NZ_FMUX01000001.1)

^c^ Putative function as thiosulfate reductase

ND: Not detected

Table S8. Enzymes involved in oxygen reduction and ROS detoxification in *Desulfoluna* strains

| Enzyme | DBB ^a^ | AA1^T b^ | MSL71^T a^ |
| --- | --- | --- | --- |
| Rubredoxin–oxygen oxidoreductase | 16300 | 101503 | 5010 |
| Cytochrome *c* oxidase | 43200–43500 | 11283–11286 | 36840–36870 |
| Cytochrome *bd* oxygen reductase | 15890–15900 | 101463–101464 | 4610–4620 |
| Superoxide dismutase | 15650 | 101439 | 4380 |
| Superoxide reductase | 40040 | 1079 | 24220 |
| Rubrerythrin | 34920 | 102199 | 19140 |
| Thiol peroxidase | 30870 | 114122 | 33350 |

^a^ Locus tag numbers are according to the genomes of strains DBB and MSL71^T^ sequenced in this study

^b^ Locus tag numbers are according to the genome of stain AA1^T^ (NZ_FMUX01000001.1)

References

1. Monserrate E, Häggblom M. Dehalogenation and biodegradation of brominated phenols and benzoic acids under iron-reducing, sulfidogenic, and methanogenic conditions. Appl Environ Microbiol 1997; 63:3911-3915.

2. Stams AJ, Van Dijk JB, Dijkema C, Plugge CM. Growth of syntrophic propionate-oxidizing bacteria with fumarate in the absence of methanogenic bacteria. Appl Environ Microbiol 1993; 59:1114-1119.

3. Bui TPN, de Vos WM, Plugge CM. *Anaerostipes rhamnosivorans* sp. nov., a human intestinal, butyrate-forming bacterium. Int J Syst Evol Microbiol 2014; 64:787-793.

4. Damsté JSS, Rijpstra WIC, Hopmans EC, Weijers JW, Foesel BU, Overmann J, et al. 13,16-Dimethyl octacosanedioic acid (*iso*-diabolic acid): A common membrane-spanning lipid of *Acidobacteria* subdivisions 1 and 3. Appl Environ Microbiol 2011; 77:4147-4154.

5. Koehorst JJ, Saccenti E, dos Santos VM, Suarez-Diez M, Schaap PJ. Expected and observed genotype complexity in prokaryotes: correlation between 16S-rRNA phylogeny and protein domain content. bioRxiv 2018:494625.

6. Koehorst JJ, van Dam JC, Saccenti E, Martins dos Santos VA, Suarez-Diez M, Schaap PJ. SAPP: functional genome annotation and analysis through a semantic framework using FAIR principles. Bioinformatics 2017; 34:1401-1403.

7. Peng P, Zheng Y, Koehorst JJ, Schaap PJ, Stams AJ, Smidt H, et al. Concurrent haloalkanoate degradation and chlorate reduction by *Pseudomonas chloritidismutans* AW-1^T^. Appl Environ Microbiol 2017; 83:00325-17.

8. Kirk DG, Palonen E, Korkeala H, Lindström M. Evaluation of normalization reference genes for RT-qPCR analysis of *spo0A* and four sporulation sigma factor genes in *Clostridium botulinum* group I strain ATCC 3502. Anaerobe 2014; 26:14-19.

9. Pfaffl MW. A new mathematical model for relative quantification in real-time RT-PCR. Nucleic Acids Res 2001; 29:2002−2007.

10. Bisaillon A, Beaudet R, Lépine F, Villemur R. Quantitative analysis of the relative transcript levels of four chlorophenol reductive dehalogenase genes in *Desulfitobacterium hafniense* PCP-1 exposed to chlorophenols. Appl Environ Microbiol 2011; 77:6261-6264.

11. Lu Y, Ramiro-Garcia J, Vandermeeren P, Herrmann S, Cichocka D, Springael D, et al. Dechlorination of three tetrachlorobenzene isomers by contaminated harbor sludge-derived enrichment cultures follows thermodynamically favorable reactions. Appl Microbiol Biotechnol 2017; 101:2589-2601.

12. Kuever J. The family *Desulfobacteraceae*. *The Prokaryotes*. Springer2014, pp 45-73.

13. Darling AE, Mau B, Perna NT. progressiveMauve: multiple genome alignment with gene gain, loss and rearrangement. PLoS One 2010; 5:e11147.

14. Hug LA, Maphosa F, Leys D, Löffler FE, Smidt H, Edwards EA, et al. Overview of organohalide-respiring bacteria and a proposal for a classification system for reductive dehalogenases. Phil Trans R Soc B 2013; 368:20120322.

15. Letunic I, Bork P. Interactive Tree Of Life v2: online annotation and display of phylogenetic trees made easy. Nucleic Acids Res 2011; 39:W475-W478.

16. Smidt H, de Vos WM. Anaerobic microbial dehalogenation. Annu Rev Microbiol 2004; 58:43-73.

17. Dobson L, Reményi I, Tusnády GE. CCTOP: a Consensus Constrained TOPology prediction web server. Nucleic Acids Res 2015; 43:W408-W412.

18. van den Bogert B, de Vos WM, Zoetendal EG, Kleerebezem M. Microarray analysis and barcoded pyrosequencing provide consistent microbial profiles depending on the source of human intestinal samples. Appl Environ Microbiol 2011; 77:2071-2080.

19. Daims H, Brühl A, Amann R, Schleifer K-H, Wagner M. The domain-specific probe EUB338 is insufficient for the detection of all Bacteria: development and evaluation of a more comprehensive probe set. Syst Appl Microbiol 1999; 22:434-444.

20. Tian L, Scholte J, Borewicz K, Bogert B, Smidt H, Scheurink AJ, et al. Effects of pectin supplementation on the fermentation patterns of different structural carbohydrates in rats. Mol Nutr Food Res 2016; 60:2256-2266.

21. Muyzer G, De Waal EC, Uitterlinden AG. Profiling of complex microbial populations by denaturing gradient gel electrophoresis analysis of polymerase chain reaction-amplified genes coding for 16S rRNA. Appl Environ Microbiol 1993; 59:695-700.

22. Moore SJ, Warren MJ. The anaerobic biosynthesis of vitamin B_12_. Biochem Soc Trans 2012; 40:581-586.
